# Introducing an Experimental Route to Identify and Unify Lab‐Scale Redox‐Flow Battery Cell Performances via Molar Fluxes and Cell Constants

**DOI:** 10.1002/smtd.202401670

**Published:** 2025-05-28

**Authors:** Sebastian Fricke, Luuk Kortekaas, Martin Winter, Mariano Grünebaum

**Affiliations:** ^1^ Helmholtz‐Institute Münster IMD‐4 Forschungszentrum Jülich GmbH Corrensstraße 48 48149 Münster Germany; ^2^ Materials Chemistry Faculty of Science and Engineering University of Groningen Groningen 9747 AG The Netherlands; ^3^ MEET Battery Research Center University of Münster Corrensstraße 46 48149 Münster Germany

**Keywords:** cross‐comparison, experimental method, mass‐transport, molar fluxes, redox‐flow battery, RFB optimization

## Abstract

Redox flow batteries (RFBs) are a promising technology for grid energy storage based on their high potential for scalability, design flexibility, high efficiency, and long durability, hence great effort has been invested in this area of research. However, due to the large differences in lab‐scale RFB cell design and construction as well their operational performance, fundamental studies on innovative RFB components (e.g., active materials, separators, additives) compare poorly due to the lack of standard setups, settings, and procedures. This work introduces an experimental calibration route for aqueous as well as nonaqueous RFBs based on a simple mass transport model using molar fluxes, enabling one to compare dissimilar lab‐scale RFB cell setups by introducing several RFB parameters: First, *K*1, which summarizes the operating parameters of an RFB to identify the critical ratio (*K*1_critical_) needed for efficient charge–discharge cycling using a simple overvoltage and charge efficiency evaluation; second, the RFB cell constant *ζ*, quantifying the influence of a lab‐scale RFB setup on its performance; and finally, *K*2, ultimately enabling full comparison of (idealized) *K*1_critical_ operating parameters across RFB cell setups.

## Graphical abstract

Introducing an experimental route to identify optimal lab‐scale redox flow batteries (RFB) operating parameters between electrolyte flow rate, redox‐active species concentration and applied current for aqueous and nonaqueous calibration electrolytes via a molar flux concept. *K*1_critical_ as an RFB‐setup performance evaluating, *ζ* as an RFB cell‐constant, and *K*2 as an cell‐architecture independent performance characteristic number are introduced to improve comparability between lab‐scale RFB architectures.

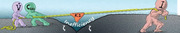

## 1 Introduction

Redox‐flow batteries (RFBs) are a highly promising grid energy storage technology due to their easy scalability, high design flexibility, high energy efficiency, as well as mechanical and electrochemical durability.^[^ ^1^, ^2^ ^]^ In recent years, considerable progress has been made in developing novel redox‐active species (RAS),^[^ ^3^, ^4^, ^5^ ^]^ electrolyte additives,^[^ ^6^, ^7^ ^]^ porous separators, and ion‐exchange membranes,^[^ ^8^, ^9^, ^10^ ^]^ as well as in furthering battery cell design (flow fields, bipolar plates, etc.) and construction,^[^ ^11^, ^12^ ^]^ with a main focus to improve performance, environmental impact and affordability of redox‐flow batteries. Additionally, a lot of work has been put into performance metrics^[^ ^13^ ^]^ and optimum cell operation conditions to press operating costs and to increase energy efficiency.

Computational simulations have proven to be a tool of choice in this area to investigate electrolyte flow behavior,^[^ ^14^, ^15^ ^]^ mass transport phenomena,^[^ ^16^, ^17^, ^18^, ^19^ ^]^ and optimum operation settings. Although simulations produce promising and practically relevant results, they are less likely to find application in the vast number (and thereby large variety) of individual RFB cell setups. In addition, each individual case requires a high level of understanding of mathematics and physics used for simulations, as well as considerable computer power for the related calculations.

In order to identify efficient operating parameters for a newly assembled lab‐scale RFB setup two steps are needed: First, finding general reproducible operating parameters for this setup; second, optimizing those operating parameters to maximum efficiency. The aim of this work is to present an easy and simple experimental route explaining the influence of operating parameters and to identify the optimal values for a given lab‐scale RFB setup, helping to tackle limitations arising from mass‐transport overvoltages. This experimental route applies constant current calibration cycling experiments using an aqueous as well as nonaqueous Fe^II+^|Fe^III+^ model electrolyte based on ferrocene/ferrocenium and ferro‐/ferricyanide, respectively, as a tool to characterize charge–discharge behavior. Additionally, a mass transport model considering an RFB as a chemical flow reactor instead of a classical battery is devised based on volume flow rates, RAS concentrations, and applied currents. These operating parameters are boiled down to productive and consumptive molar fluxes ${\dot{n}}_{p}$ and ${\dot{n}}_{c}$, respectively, with their ratio (*K*1) being a vital parameter for charge–discharge cycling and optimal cycling (*K*1_critical_) conditions. Furthermore, we present an experimental route based on electrochemical impedance spectroscopy (EIS) and on an ISO 7888 calibrating standard for enabling comparison between different RFB geometries and their efficiency ranges, introducing an RFB cell constant (*ζ*) and a second newly introduced RFB parameter *K*2.

## 2 The Redox‐Flow Battery as Both Electrochemical Device and Flow‐Reactor: Productive versus Consumptive Molar Fluxes

As known from literature, RFB operating parameters (i.e., active material concentration, electrolyte flow rate, and applied electrical current) have a significant impact on charge–discharge behavior and performance.^[^ ^18^, ^20^ ^]^ Hence, optimum operating parameters are specific to individual RFB setups. This work introduces RFB setup‐specific parameters that, through a systematical step‐by‐step optimization process, enable comparison between different RFB setups. The beginner‐friendly experimental route focusses explicitly on galvanostatic charge–discharge measurements on the individual RFB setup without the need for computational simulations, finding a balance between operating parameters from the perspective of both a flow reactor as well as an electrochemical device.

### 2.1 Introducing a Fundamental RFB Operating Parameter and Molar Fluxes

A lab‐scale RFB cell, meaning here a single assembly of two electrodes separated by electrolyte and a membrane or separator, can be considered a special type of flow reactor, where a reaction mixture (an electrolyte solution consisting of solvent, conducting salt/charge balancing species, redox active species (RAS), and possible additives) is converted in a controlled electrochemical redox event) inside the reactor (RFB cell) principally through mass transport. Therefore, a productive molar flux (${\dot{n}}_{p}/\mathrm{mol}\;s^{-1})$, determining the amount of RAS available at the flow‐through reactor surface per unit of time, and a consumptive molar flux (${\dot{n}}_{c}/\mathrm{mol}\;s^{-1}$), describing the reaction rate of the RAS to the charged species, are introduced here. ${\dot{n}}_{p}$ depends on the volume flow rate $(\dot{V}/L\;s^{-1}$), the RAS concentration (*c*_RAS_/mol L^−1^), and the number of electrons transferred during the reactions (*z*, for the Fe^II+^|Fe^III+^ calibrations electrolyte used here *z* = 1), according to Equation (1). ${\dot{n}}_{c}$ is based on the Faradaic law calculated by the applied electric current (*I*/*A*) using the Faraday constant *F* ($=96\;485/A\;s\;\mathrm{mo}l^{-1})$ and *z*, according to Equation (2)

(1) $${\dot{n}}_{p}=z\cdot c_{\mathrm{RAS}}\cdot \dot{V}$$

(2) $${\dot{n}}_{c}=I/\left(z\cdot F\right)$$

Essentially, ${\dot{n}}_{p}$ determines how many moles of RAS are present at the electrode surface per unit of time, while ${\dot{n}}_{c}$ determines how many moles thereof are electrochemically converted and how many charge balancing species will pass through the separator as a result per unit of time. Note that ${\dot{n}}_{c}$ defines the reaction rate of the RFB reactor by the total number of electrons transferred through the applied current, thus the current density, a conventional parameter for RFBs, would be unsuitable for this experimental evaluation route.

### 2.2 Overview and Use of the *K*1 Ratio

The stoichiometric number (*λ*) has previously been used to describe the ratio of real consumed charge relative to the theoretically available charge based on Faradaic law.^[^ ^21^ ^]^ From varying operating conditions, it is established that the relationship between the operating parameters $\dot{V}$, *c*_RAS_ and *I* has a great impact on the effectiveness of RFB cycling. Considering the influence of the operating parameters from a mass transport perspective, the ratio of productive and consumptive molar flux, *K*1, can be determined through Equation (3)

(3) $$\begin{array}{c} K1={\dot{n}}_{p}/{\dot{n}}_{c}\;=\left(z\cdot F\cdot c_{\mathrm{RAS}}\cdot \dot{V}\right)/I \end{array}$$

With the applied operating parameters summarized in a single *K*1 value, we will show that the normalized charge efficiency (*NCE*/%) and the overvoltage (*η*/V) can be determined for specific *K*1 values through the experimentally measured charging capacity (*Q*_c. exp._/mAh), the theoretical maximum capacity by RAS concentration (*Q*_c. theo._/mAh) and the mean charge/discharge cell voltage (${\bar{U}}_{\mathrm{charge}}$/V and ${\bar{U}}_{\mathrm{discharge}}$/V)

(4) $$NCE=(Q_{c.\;\exp.}/Q_{c.\mathrm{theo}.})\cdot 100$$

(5) $$\eta =(\left|{\bar{U}}_{\mathrm{discharge}}\right|+{\bar{U}}_{\mathrm{charge}})/2$$

Ultimately, plotting each *K*1 ratio against their achieved *NCE* and *η* then enables an overall performance evaluation of the individual RFB setup, to find the minimum critical *K*1 ratio for efficient (dis)charge cycling.

### 2.3 Introduction of RFB Specific, Minimum Operating Parameter Ratio *K*1_critical_

Considering the efficiency evaluation, it becomes clear that for every individual RFB setup there is a specific and fixed critical *K*1 ratio (*K*1_critical_) above which the charge–discharge cycling becomes sufficiently efficient by eliminating mass transport limitations. In this work, we define that a minimum efficiency criterion is reached at 10% over minimum overvoltage (110% of *η*_min_). This 10% ensures a minimum overvoltage level for efficient cycling, without spending energy on an unnecessarily high molar flux (via excessive electrolyte pumping at even higher *K*1 values), while simultaneously not losing energy on a unnecessarily high overvoltage (through a rapidly decreasing presence of RAS in the reactive space of the electrode surface at lower *K*1 values). *K*1_critical_ is, thus, calculated according to Equation (6)

(6) $$K1_{\mathrm{critical}}=K1\left(\;at\;\eta_{\min}\cdot 1.10\;\right)$$

*K*1_critical_ thus serves, first, to determine an efficient operating parameter ratio of ${\dot{n}}_{p}$ and ${\dot{n}}_{c}$ for an individual RFB setup, and second, to have a comparative benchmark value between similar RFB setups. If the *K*1_critical_ value of an individual RFB setup is lower compared to another, it exhibits a higher energy efficiency, for example due to better diffusive mass transport, provided that all other RFB components have remained constant (e.g., membrane/separator, carbon felt). Additionally, if the RFB setup remains constant, the cycling performance of different membranes/separators or carbon felts can be compared. Generally, lower *K*1_critical_ values achieved for an individual RFB setup therefore mean better cycling performance.

### 2.4 Limiting Cases of *K*1

**Figure 1:**
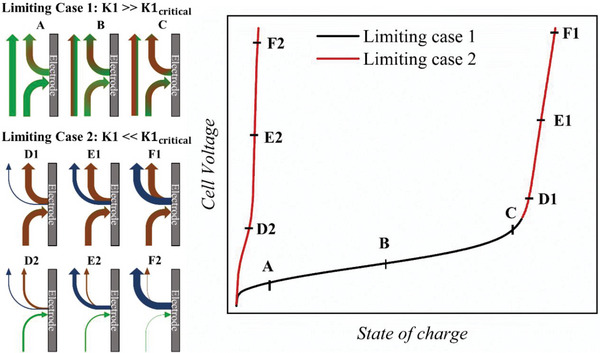
(left) Schematic illustration of the RAS molar fluxes (green: fresh electrolyte, red: converted electrolyte, blue: parasitic side reactions) and their quantities (thickness of the arrows) during a charging process for limiting case 1 (A–C, *K*1 ≫ *K*1_critical_) and limiting case 2 (*K*1 ≪ *K*1_critical_, D1 to F1 for tipping *K*1≪ *K*1_critical_ at high SoC and D2 to F2 for already *K*1 ≪ *K*1_critical_ at beginning of charge/discharge). Note that the shortage of fresh RAS that occurs at high SoC resembles that when the charging process starts off at too low ${\dot{n}}_{p}$; (right) An idealized polarization curve with the different stages (A–F) during a charging process corresponding to the RAS molar fluxes.

When applying a range of *K*1 ratios for charge–discharge experiments of a RFB setup, this ultimately yields the following two border cases: 1) *K*1 ≫ *K*1_critical_ and 2) *K*1 ≪ *K*1_critical_. Since the basic potential curve profile with its voltage ranges (electrochemical kinetic losses at the beginning, ohmic losses during the charging plateau, and mass‐transport losses at the voltage‐run off) is already known from the literature,^[^ ^21^ ^]^ the following section will act to rationalize the two limiting cases (**Figure** **1**).

For the limiting case 1, starting charge–discharge cycling with a *K*1 ratio larger than *K*1_critical_, the productive RAS supply ${\dot{n}}_{p}$ is considerably higher than the consumptive ${\dot{n}}_{c}$, offering an oversupply of RAS. Thus, only a fraction of the RAS passing by the electrode is electrochemically converted (Figure 1, point A). As cycling progresses and the SoC and cell voltage rise during the ohmic loss plateau, the concentration of unreacted RAS, and thus also the *K*1 ratio, steadily decreases (Figure 1, point B). At some point, the RAS concentration will approach the theoretical minimum to supply enough charge carriers for the constant current to pass the electrode surface, leading to a mass‐transport limiting voltage‐run off at the end of the charging step as the *K*1 value drops below *K*1_critical_ (Figure 1, point C to D1). From this point onward, the model switches to limiting case 2 until the current is reversed.

For the limiting case 2, starting to (dis)charge with a *K*1 ratio lower than *K*1_critical_, the productive ${\dot{n}}_{p}$ is lower than the consumptive ${\dot{n}}_{c}$, i.e., the amount of fresh RAS transported to the electrode surface is short of the applied consumptive ${\dot{n}}_{c}$ (Figure 1, point D2). This lack of fresh RAS is analogous to the final stage of electrochemical charge/discharge at mass‐transport limiting voltage‐run off, as the amount of unreacted RAS decreases over time until it cannot sustain the demanded consumptive molar flux ${\dot{n}}_{c}$ (Figure 1, point D1 through F1). Essentially, if the initial *K*1 ratio has already been set to a smaller *K*1_critical_ value, the potential curve skips the ohmic loss voltage plateau and tips directly into the mass‐transport limiting voltage‐run off (Figure 1 D2 through F2). As side reactions are not only possible but even required to achieve the demanded consumptive molar flux, an additional molar flux representing side reactions (Figure 1 blue arrows) was added. In either situation, whether limiting case 2 occurs at the end of a charging step or directly at the beginning, this limiting case can be described by a strongly lacking concentration of RAS. Thus in our model, the diffusional mass transport toward the electrode surface area can be described by fick’s second law.^[^ ^22^ ^]^ Notably, the lack of fresh RAS is much more evident at high SoC than at low SoC, making the *K*1 value a dynamic parameter that changes during cycling, e.g., at 99% SoC an efficient *K*1 requires dramatically higher flow rates from that at 0% SoC. After all, *K*1 decreases while (dis)charging as the still available unreacted *c*_RAS_ in the numerator of the equation ($K1=(z\cdot F\cdot c_{\mathrm{RAS}}\cdot \dot{V})/I$) continuously decreases, effectively making the critical tipping point to the mass‐transport limited voltage run‐off dependent on the preset constant flow rate. To counteract this, different dynamic flow rate control strategies have already been developed, which adapt the electrolyte flow rate to the respective SoC.^[^ ^23^, ^24^ ^]^ This ensures that the optimized balance between systemic energy efficiency and overvoltage is preserved throughout the charge/discharge cycle, until a certain cut‐off flow rate is reached.

### 2.5 Introduction of RFB Cell Constant *ζ*

In order to rule out the variation of optimum parameters between varying cell‐setups and electrolyte chemistries, additionally the RFB cell constant *ζ* can be defined according to standard electrochemical impedance spectroscopy (EIS). This cell geometric factor is calculated by determining the bulk resistance (*R_Ω_*/Ω) of an RFB setup via ISO 7888 standard (i.e., EIS using a 0.1 m KCl aqueous electrolyte with fixed specific conductivity of *σ*_ISO7888_ = 0.0129 S cm^−1^ or the newly proposed nonaqueous reference supporting electrolyte consisting of 0.3 m TBABF_4_ and acetonitrile *σ*_ACN + 0.3 M TBABF4_ = 0.022 S cm^−1^) according to Equation (7)

(7) $$\begin{array}{c} \zeta =\mathrm{specific}\;\mathrm{conductivity}/\mathrm{absolute}\;\mathrm{conductivity}=\sigma /\Sigma =\sigma \cdot R_{\Omega }\;\\ \zeta =\mathrm{inter}\;\mathrm{electrode}\;\mathrm{distance}/\mathrm{electrochemical}\;\mathrm{active}\;\mathrm{area}=d/A \end{array}$$

Importantly, the value of *ζ* is dependent on cell setup in its entirety and will differ from cell to cell in various redox flow setups, as it is calculated via the conductivity arising from the combined areal‐ and inter‐electrode geometry (if both are doubled, *ζ* remains unchanged). It is exactly this difference that will reflect a discrepancy in effectivity matching the situational conductivity (exact combined surface area and electrode spacing that changes with every cell built), making the metric a very attractive candidate for determining general cell conduction properties. *ζ* thus, in addition to *K*1_critical_, not only summarizes the (dis)charging performances in a parameter but also the cell geometric structures of the electrochemically active components of the RFB structure (i.e., carbon felt, flow field, bipolar plate, membrane). Using the defined standard electrolytes for aqueous and nonaqueous electrolytes as well as the experimental route (explained in Section 3), the influences of different electrochemically active components can be quantified and thus made comparable. Small cell constants are desirable, for when *ζ* is smaller for one RFB setup than another, the superior conductivity causes lower setup‐related ohmic losses while (dis)charging.

### 2.6 Introduction of RFB Setup Independent Minimum Operating Parameter Ratio *K*2

To eliminate the influence of cell geometry on cell performance, the constant *K*1_critical_ can simply be divided by the cell constant *ζ* yielding the constant *K*2 and is calculated according to Equation (8)

(8) $$K2=K1_{\mathrm{critical}}/\zeta$$

*K*2 thus acts as a combination of the charge–discharge performance comparison parameter *K*1_critical_ and the cell‐geometric parameter, thereby reflecting cell setup‐dependent performance and enabling comparison between different RFB structures.

## 3 Experimental Route to Determine *K*1_critical_ via *K*1 Evaluation and *ζ* and *K*2 via ISO 7888

*K*1, i.e., the ratio between ${\dot{n}}_{p}$ and ${\dot{n}}_{c}$, summarizes the overall operating parameters of the lab‐scale RFB and can be used to describe the expected energy efficiency and RAS utilization of the cycling process: a high *K*1 should result in high material utilization and cycling efficiency, and a low *K*1 corresponds to a low utilization and cycling efficiency. Through systematic variation of *K*1 by adjusting *c*_RAS_, $\dot{V}$ and *I*, the critical minimum for efficient operating parameters, *K*1_critical_, of an individual RFB structure can be determined. The simple experimental route developed for this is based on galvanostatic charge–discharge experiments using an aqueous electrolyte (K_4_[Fe^II^(CN)_6_]|K_3_[Fe^III^(CN)_6_] with 0.1 m KCl in deionised water) as well as a nonaqueous electrolyte (ferrocene|ferrocenium (Fc|FcBF_4_), as proposed by Armstrong and coworkers.^[^ ^24^ ^]^) As Fe^II^|Fe^III^ shows a well‐known, highly reversible and fast redox behavior, this redox‐pair has been broadly applied as standard electrolyte also in cyclic voltammetry experiments.^[^ ^25^ ^]^ Therefore, although these 0 V cell voltage symmetric RAS would not be practical for “real” RFB applications, the stable and established Fe^II^|Fe^III^ electrolyte is well suited as calibration material within the mass‐transport model presented here.^[^ ^26^, ^27^ ^]^ In the experimental route, a previously published lab‐scale RFB setup (Figure S1, Supporting Information)^[^ ^28^ ^]^ was used to perform all of the below mentioned parameter determination and optimization (electrochemical active area 2.4 cm^2^).

First, the effect of varying the productive flow (i.e., by either increasing RAS concentration or flow rate of the here used nonaqueous Fc|FcBF_4_ standard calibration electrolyte) on the impedance of the cell is investigated (Section 4). The bulk resistances that are later used to calculate the cell constant were determined using EIS for the ISO 7888 standard in the aqueous and the nonaqueous standard (0.3 m TBABF_4_ in acetonitrile). Second, RAS concentrations, electrolyte volume flow rates, and electrical currents are systematically varied in galvanostatic cycling experiments so that an operating parameter matrix of at least 3 × 3 × 3 is created and a broad *K*1 window is covered. The aqueous and nonaqueous standard calibration electrolytes, therefore, are galvanostatically cycled at four or three different electrical currents each (20, 30, 40, and 60 mA for the aqueous electrolyte and 5, 10, and 15 mA for the nonaqueous), at three different RAS concentrations (50, 100, and 150 mm of K_4_[Fe^II^(CN)_6_]|K_3_[Fe^III^(CN)_6_] for aqueous and 5, 10, and 15 mm of Fc|FcBF_4_ for nonaqueous) and at three or four different flow rates (5, 10, and 20 mL min^−1^ for aqueous and 5, 10, 20, and 30 mL min^−1^ nonaqueous) to determine the effect of varying the compositional ratio of *K*1 on cycling performance (Section 5). For each charge–discharge cycle the *K*1 value is calculated via the corresponding ${\dot{n}}_{p}$ and ${\dot{n}}_{c}$ using Equation (3) and the resulting normalized charge efficiency (*NCE*/%) and the overvoltage (*η*/V) are then determined using Equations (4) and (5), which allows determination of the RFB structure‐specific *K*1_critical_ (Section 6). Third, to determine the cell constant *ζ* through Equation (7), the bulk resistance of the respective RFB structure is extracted from the EIS data (Section 7). Finally, the combined parameter *K*2 is calculated for the lab‐scale RFB setup used here from the determined RFB setup‐specific parameters *K*1_critical_ and *ζ*, serving as initial comparative value for other lab‐scale RFB setups.

**Figure 2:**
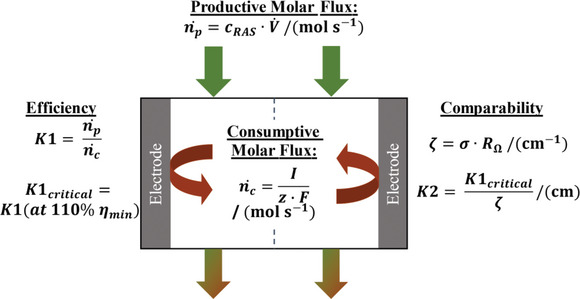
Schematic illustration of productive (${\dot{n}}_{p})$ and consumptive (${\dot{n}}_{c}$) molar fluxes, the related operating parameter *K*1, its efficiency evaluation value *K*1_critical_, the RFB cell constant *ζ*, and the setup‐independent performance parameter *K*2.

For a comprehensive overview, **Figure** **2**; and Table S1 (Supporting Information) list all newly introduced experimental metrics to evaluate the RFB setup performance using the concept of molar fluxes.

## 4 Impact of Productive Molar Flux on Mass‐Transport Resistances

Impedance spectroscopy is a bulk technique that maps the overall cell impedance, the reciprocal of conductance. Although every single cell component that is involved in the electric circuit contributes to the overall impedance of a battery, not all contribute in a significant manner and many overlap as well, making it hard to resolve individual processes.^[^ ^29^, ^30^ ^]^ Although a systematic assessment of cell setup stages can elucidate the nature of impedance contributions from different components, it is the overall impedance that ultimately governs cycling efficiency. Therefore, symmetrical RFBs are often modeled by clustering circuit components into a bulk resistance (*R_Ω_*/Ω) and a single mass‐ and electron‐transfer related parallel component for the diffusion boundary layer that consists of the charge‐transfer resistance at the porous electrode surface (*R*_1_/Ω) and a Warburg element (*W*) for the mass‐transport on the one side, and a constant‐phase element (*CPE*) that mimics the imperfect double layer capacitance on the other.^[^ ^31^, ^32^ ^]^ Although this is an accurate approximation under static conditions, especially the mass‐transfer component is influenced by flow conditions, one of the perks of redox‐flow batteries.^[^ ^29^ ^]^ Under flow conditions, the effective thickness of the diffusion layer that is integral to mass‐transport kinetics is evidently reduced, as the Warburg impedance transitions from the classical ≈45° angle Warburg tail (i.e., containing a perfectly split increase in capacitive and resistive contribution over increasing frequencies) to a finite‐length Warburg model.^[^ ^33^ ^]^ Indeed, such a response of the Warburg impedance is also observed in conventional electrochemical cells using carbon‐black modified rotating disc electrodes, where the Warburg component at the porous electrode surface is reduced to a parallel finite‐length RC component upon increasing rotational velocities.^[^ ^34^, ^35^ ^]^

**Figure 3:**
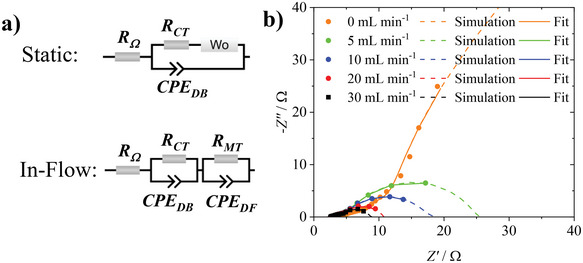
a) Equivalent circuit models used to evaluate the impedance spectroscopic data. The classical Warburg impedance, that is observed under static conditions, gets reduced to a finite‐length Warburg element under in‐flow conditions. b) EIS raw data evaluation (dots) including the fitted function (continuous line, 0.01 Hz to 1 MHz) and the simulation for lower frequencies (dashed line, 0.01 mHz to 1 MHz) of 0.3 m TBABF_4_ in acetonitrile supporting electrolyte with 10 mm Fc|FcBF_4_ at flow rates between 5 and 30 mL min^−1^. The EIS evaluation for 5 and 15 mm can be found in Figure S2a) and b) (Supporting Information), respectively.

Here, to identify the impact of ${\dot{n}}_{p}$ on the overall cell resistance, EIS measurements are carried out using the nonaqueous Fc|FcBF_4_ standard calibration electrolyte at different RAS concentrations (5–15 mm) and volume flow rates (0–30 mL min^−1^). Two different models will be applied, one including the classical Warburg element pertaining to a static RFB, and one including a finite‐length Warburg element represented by an additional parallel RC component for in‐flow impedance measurements (**Figure** **3**a).

**Figure 4:**
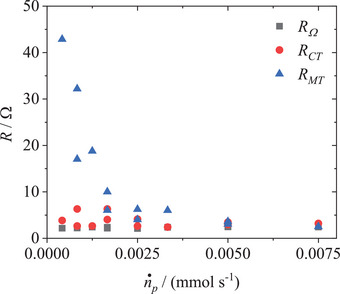
Productive molar fluxes (${\dot{n}}_{p}$/(mmol s^−1^)) plotted against the different evaluated resistances: bulk resistance (*R_Ω_*/Ω), charge‐transfer resistance inside the double layer (*R*_CT_/Ω) and the mass‐transport resistance in the diffusive layer (*R*_MT_/Ω) using the equivalent circuit shown in Figure 3a. Table S2 (Supporting Information) summarizes all measured resistances plotted here from the EIS raw data (Figure 3b; and Figure S2a) and b), Supporting Information).

Under static conditions, the classical Warburg tail is observed as expected, signifying that diffusive mass‐transport in the bulk of the solution is strongly limiting the conductivity in the low frequency range (Figure 3b), orange line)). In line with previous reports, the Warburg impedance reduces to a finite‐length model upon increasing the flow rate, eventually reaching an apparent optimum at higher flow rates.^[^ ^29^, ^34^, ^35^ ^]^ The concentration of RAS affects how quickly the impedance is reduced upon increasing the flow rate, with higher concentrations of active species being more capable of balancing out in the stagnant diffuse layer (Figure S2 a) and b), Supporting Information). Independent of concentration, at high enough flow rates the convection is at a near‐maximum, and increasing the flow rate further will do increasingly less to effectively reduce the size of the diffusion layer through which the RAS travels to the well‐mixed bulk (analogous to the levelling off of efficiencies below in Section 6). Considering the *x*‐axis intercepts of the impedance spectra, all three resistance regions summed up will eventually affect the observed overpotential that is required for the reaction to proceed, with the mass‐transfer resistance arising from Warburg impedance being inversely dependent on the flow rate. Consequently, most relevant to the performance assessment are the data at higher flow rates, and in particular the left‐hand side *x*‐axis intercept at high frequency for determining the Ohmic resistance (*R_Ω_*/Ω), the mid‐frequency *x*‐axis intercept for determining the charge‐transfer resistance at the electrode surface (*R*_CT_/Ω) and the right‐hand side *x*‐axis intercept for determining the mass‐transfer resistance of the RAS within the diffusion layer of the electrode pores (*R*_MT_/Ω) (**Figure** **4**; and Table S2, Supporting Information). As expected, *R_Ω_* and *R*_CT_ are observed to behave independently of the flow rate and RAS concentration due to the unchanged cell structure and material composition, while *R*_MT_ levels off at higher flow rates and does so quicker at higher concentrations (i.e., larger *K*1 values).

## 5 Impact of Molar Fluxes and *K*1 on Charge–Discharge Performance

To illustrate the influence of productive (${\dot{n}}_{p}$ consisting of *c*_RAS_ and $\dot{V}$) and consumptive (${\dot{n}}_{c}$ consisting of *I*) molar fluxes on the charge–discharge polarisation behavior in the lab‐scale RFB setup example, Figure 4 compares systematically different applied RFB operating parameters. Of the nonaqueous Fc|FcBF_4_ standard calibration electrolyte, the *c*_RAS_, $\dot{V}$, and *I* and, thus, the ratio of ${\dot{n}}_{p}$ to ${\dot{n}}_{c}$, are varied one parameter at a time while keeping the other two constant (for the nonaqueous electrolyte see Figures S3–S5, Supporting Information). Table S7 (Supporting Information) summarizes the resulting *η* and *NCE* for each nonaqueous *K*1 ratio; aqueous K_4_[Fe^II^(CN)_6_]|K_3_[Fe^III^(CN)_6_] electrolyte polarisation curves are shown in Figures S7–S9 (Supporting Information) and the resulting *η* and *NCE* for each aqueous *K*1 ratio are summarized in Table S8 (Supporting Information).

**Figure 5:**
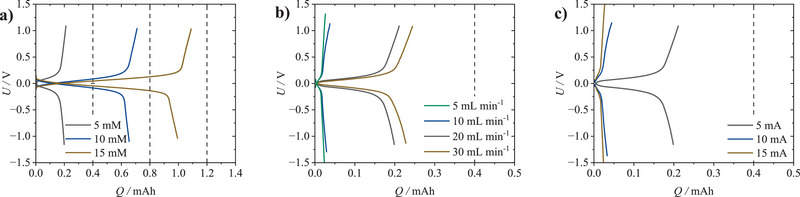
Impact of productive and consumptive molar fluxes on galvanostatic charge–discharge polarization of the nonaqueous Fc|FcBF_4_ electrolyte, through systematic variation of *c*_RAS_, $\dot{V}$ and *I* (for quantitative efficiency evaluation compare Table S7 (Supporting Information); for aqueous electrolyte polarization curves see Figures S7–S9 and Table S8, Supporting Information): a) variation of Fc|FcBF_4_ RAS concentration (*c*_RAS _= 5, 10, 15 mm) at constant volume flow rate of 20 mL min^−1^ and current of 5 mA (${\dot{n}}_{p}$ = 0.0017, 0.0034, and 0.005 mmol s^−1^; ${\dot{n}}_{c}$ = 0.000 052 mmol s^−1^; *K*1 = 32, 64, and 96). b) Variation of volume flow rate ($\dot{V}$ = 5, 10, 20, and 30 mL min^−1^) at constant RAS concentration of 5 mm and current of 5 mA (${\dot{n}}_{p}$ = 0.00 042, 0.00 083, 0.0017, and 0.0025 mmol s^−1^; ${\dot{n}}_{c}$ = 0.000 052 mmol s^−1^; *K*1 = 8, 16, 32, and 48) and c) variation of current (*I* = 5, 10, and 15 mA) at constant concentration of 5 mm and volume flow rate of 20 mL min^−1^ (${\dot{n}}_{p}$ = 0.0017 mmol s^−1^; ${\dot{n}}_{c}$ = 0.000 052, 0.0001, and 0.00 016 mmol s^−1^; *K*1 = 32, 16, and 11).

Higher *K*1, via increasing the productive molar flux at elevated *c*_RAS_ (**Figure** **5**a; *K*1 = 32, 64, and 96), not only increases the relative *NCE* (53%, 88%, and 91%, respectively) but also reduces *η* (0.21, 0.11, and 0.1 V, respectively), thus, overall resulting in a better (dis)charge performance. Similar results are achieved when comparing the $\dot{V}$ variation (Figure 5b; *K*1 = 8, 16, 32, and 48), as for higher $\dot{V}$ the *NCE* (6%, 9%, 53%, and 61%, respectively) increases and *η* (0.39, 0.36, 0.21, and 0.19 V, respectively) decreases as well. Similarly, a decrease in the applied *K*1 ratio via increased current *I* (Figure 5c; *K*1 = 32, 16, and 11) also decreases the *NCE* (53%, 15%, and 8%, respectively) and increases *η* (0.21, 0.49, and 0.92 V, respectively).

Overall, the *K*1 increases with higher *c*_RAS_, $\dot{V},$ or lower *I* by either directly improving the RAS mass‐transport in case of the first two operating parameters, or lowering the overall current demand of fresh RAS in case of the last, to ultimately cause drastic improvement in (dis)charge performance, as expected.^[^ ^36^ ^]^ Importantly, the general consistency of the methodology introduced here is clearly reflected by the similar *K*1 ratios composed of different *c*_RAS_, $\dot{V},$ and I, as the resulting *NCE* (*NCE*(*K*1 = 32) = 63% ± 5%, *NCE*(*K*1 = 48) = 65% ± 5%), and *η* (*η*(*K*1 = 32) = 0.21 ± 0.02 V, *η*(*K*1 = 48) = 0.15 ± 0.02 V) remain nearly constant for the nonaqueous standard electrolyte (compare Table S6, Supporting Information).

## 6 Identifying of RFB Specific, Minimum Operating Parameter Ratio *K*1_critical_

**Figure 6:**
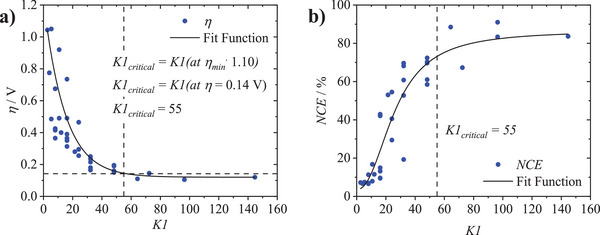
a) Overvoltage (*η*/V) and b) normalized charge efficiency (*NCE*/%) plotted against the different *K*1 values applied for the nonaqueous Fc/FcBF_4_ electrolyte, here *η*_min, nonaqueoues_ = 0.13 V and a *NCE*_max, nonaqueoues_ = 83% resulting with Equation (6) in a *K*1_critical, nonaq._ = 55 (at $\eta_{K1_{\mathrm{critical},\;\mathrm{nonaq}.}}$ = 0.14 V). For aqueous [Fe^III^(CN)_6_]^3−^ / [Fe^II^(CN)_6_]^4−^ electrolyte *η*_min, aqueous_ = 0.14 V and *NCE*_max, aqueoues_ = 82% were achieved (Figure S10 (Supporting Information); $\eta_{K1_{\mathrm{critical},\;\mathrm{aq}.}}$= 0.16 V) resulting in a *K*1_critical, aq._ = 66. Table S7 (Supporting Information) additionally summarizes all calculated overvoltage and *NCE* values for each nonaqueous and Table S8 (Supporting Information) for each aqueous *K*1.

In line with the molar flux mass‐transport perspective, the strong exponential dependence of the cycling efficiency on the operating parameters is given by *K*1 ratios used either corresponding to limiting case 1 (*K*1 ≫ *K*1_critical_) or limiting case 2 (*K*1 ≪ *K*1_critical_). For an exact determination of the limiting case boundary, i.e., *K*1_critical_, for the lab‐scale RFB set‐up, **Figure** **6** plots all determined *NCE* and *η* efficiencies of the respective *K*1 ratios for the nonaqueous electrolyte (summarized in Table S7 (Supporting Information); for the aqueous *K*1 evaluation compare Figure S10 and Table S8, Supporting Information).

In line with the trend observed in impedance, the overvoltage and *NCE* parameters show a limit to their reduction and growth, respectively, for increasing values of *K*1. The larger *K*1, the lower the mass‐transport‐induced overvoltage and thus the higher the efficiency. At very high *K*1, the overvoltage for the nonaqueous electrolyte decreases to a minimum limit of *η*_min, nonaqueoues_ = 0.13 V (Figure 6a) and a *NCE*_max, nonaqueoues_ = 83% (Figure 6b), while the aqueous electrolyte exhibited a *η*_min, aqueous_ = 0.14 V (Figure S10a, Supporting Information) and *NCE*_max, aqueoues_ = 82% (Figure S10b, Supporting Information), respectively. In this high *K*1 range, an efficiency maximum is reached due to limiting factors presumably based on ohmic losses due to electrolyte composition (e.g., rate of electron transfer, solubility of the charged species) and RFB cell geometry, so that an improvement of the RAS mass transport no longer leads to an improvement in efficiency. As presented in our molar flux transport perspective, only *K*1 values greater than *K*1_critical_ (limiting case 1) have the productive mass transport ${\dot{n}}_{p}$ of the RAS to the electrode surface requested by the consumptive ${\dot{n}}_{c}$. If the applied *K*1 is lower than *K*1_critical_ (limiting case 2), the lack of RAS at the electrode surface for electrochemical conversion, due to the insufficient ${\dot{n}}_{p}$ in relation to ${\dot{n}}_{c}$, causes a mass‐transport limited low operating efficiency. *η* is therefore higher and the *NCE* lower. To calculate *K*1_critical_, Equation (9) is used with a *η*_min, nonaqueoues_ = 0.13 V for the nonaqueous and *η*_min, aqueous_ = 0.14 V for the aqueous electrolyte

(9) $$\begin{array}{c} \eta_{K1_{\mathrm{critical},\mathrm{non}\;\mathrm{aq}.}}=\eta_{\min,\mathrm{nonaq}.}\cdot 1.10=0.13\;\;V\cdot 1.1=0.14\;V\\ \eta_{K1_{\mathrm{critical},\mathrm{aq}.}}=\eta_{\min,\mathrm{aq}.}\cdot 1.10=0.14\;V\cdot 1.1=0.16\;V\\ K1_{\mathrm{critical},\mathrm{nonaq}.}=K1\left(\;\eta_{K1_{\mathrm{critical},\mathrm{non}\;\mathrm{aq}.}}\;\right)=K1\left(0.14\;\;V\right)=55\\ K1_{\mathrm{critical},\mathrm{aq}.}=K1\left(\;\eta_{K1_{\mathrm{critical},\mathrm{aq}.}}\;\right)=K1\left(0.16\;\;V\right)=66 \end{array}$$

A value of 10% above the minimum overpotential was selected to retain high efficiency in the charge‐transfer process (i.e., the rise in *K*1 does increasingly less to lower the overpotential and increase the normalized charge efficiency), while steering clear from high overpotentials on the other end of the exponential curves. The latter also ensures that only at large Ohmic resistances (i.e., raising the minimum overpotential plateau) the *K*1_critical_ value would be significantly altered, when a 10% increase lifts the *K*1_critical_ much higher up the curve. For the exemplarily evaluated lab‐scale RFB setup presented in this work, the 110% overpotential value yields a *K*1_critical_ of 55 for the nonaqueous Fc|FcBF_4_ and of 66 for the aqueous K_4_[Fe^II^(CN)_6_]|K_3_[Fe^III^(CN)_6_] standard calibration electrolytes. The comparison of the aqueous and nonaqueous electrolytes shows no significant differences in the limit overvoltages and *NCE*s at very high *K*1 and the resulting *K*1_critical_, although different redox chemistries, electrolytes, and supporting electrolytes as well as separators (differences detailed in the Experimental Section) were used in the comparison of these two experiments. This result, in overlapping *K*1_critical_ of different electrolyte chemistries and slight setup differences, reinforces the conclusion that the *K*1_critical_ value is mainly defined by the RFB cell geometry and the resulting electrolyte flow behavior and thus also justifies *K*1_critical_ as an RFB structure‐comparative parameter.

**Figure 7:**
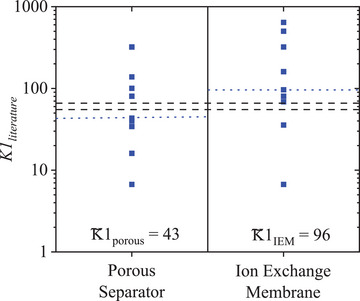
Literature calculated *K*1 values of aqueous and nonaqueous electrolytes for porous separators and ion exchange membranes (Table S9, Supporting Information) and their corresponding median: ${\tilde{K1}}_{\mathrm{porous}}$ and ${\tilde{K1}}_{\mathrm{IEM}}$ (blue dashed lines). The two black dashed lines show the range between *K*1_critical_ of 55 and 66 achieved for the lab‐scale RFB setup used in this example efficiency evaluation.

A review of reported aqueous and nonaqueous literature lab‐scale charge–discharge cycling experiments shown in **Figure** **7**; and Table S9 (Supporting Information) results in a median of a ${\tilde{K1}}_{\mathrm{porous}}$ = 43 for porous separators and a ${\tilde{K1}}_{\mathrm{IEM}}$ = 96 for the more resistive ion exchange membranes (IEM), being similar to the *K*1_critical_ at 55 (or 66) measured here with a porous separator. The higher *K*1 for IEMs could be due to the higher ionic resistivity and hence higher a compensative molar flux is needed. Notably, the wide variation within the literature *K*1 data underlines the need for a unified molar flux concept as presented here. Even though, the standard calibration electrolytes does not serve as a suitable RAS for real RFB experiments, this overlap with the literature research shows that for any lab‐scale RFB setup a *K*1 of 55 for nonaqueous or 66 for aqueous electrolyte could serve as a suitable initial ratio of productive and consumptive molar fluxes for RFB charge–discharge experiments. On this basis, more in‐depth optimisation of the operating parameters can then be carried out depending on the RFB setup (see Section 7).

As *K*1 describes the ratio between productive and consumptive molar fluxes, ${\dot{n}}_{p}$ needs to be ≈55 (or 66) times higher than ${\dot{n}}_{c}$ to run the cycling for this system in an efficient manner, which is also in the range of the literature review *K*1 median (${\tilde{K1}}_{\mathrm{porous}}$ = 43 and a ${\tilde{K1}}_{\mathrm{IEM}}$ = 96). It also becomes apparent that the same *K*1 values, which are calculated by combining different *c*_RAS_, $\dot{V}$, and *I* values, achieve similar overall overvoltage and *NCE* conductance values, which supports the molar flux balancing mass transport perspective for an efficient RFB charge–discharge cycling presented here. Overall, this efficiency evaluation, exemplified

## 7 Identifying the RFB Cell Constant *ζ*

To facilitate comparison between varying cell‐setups and electrolyte chemistries, an RFB cell constant *ζ* is introduced, analogous to conductivity measurements. This cell geometric factor *ζ* can be determined experimentally according to the ISO 7888 standard for aqueous systems (0.1 m KCl in H_2_O with a fixed absolute conductivity of *σ*_ISO7888_ = 0.0129 S cm^−1^) or the Fc|FcBF_4_ standard calibration electrolyte with the 0.3 m TBABF_4_ in acetonitrile supporting electrolyte for nonaqueous systems. *ζ* is simply calculated by experimentally determining the bulk resistance (*R_Ω_*, the high‐frequency *x*‐axis intercept in the EIS spectrum) of the RFB cell with the respective calibration electrolyte using EIS via Equation (5).

For illustration the cell constants of a coaxial EIS reference cell from Grünebaum et al. and different RFB construction stages were compared.^[^ ^37^ ^]^ A detailed explanation of the impedance measurements, as well as the data and the corresponding cell constant calculation is shown in the Supporting Information (**Table** **1**).

**Table 1:** Experimentally calculated cell constants based on the measured bulk resistances of the different setup stages using the specific conductivity of the ISO 7888 aqueous reference electrolyte (*σ*_ISO7888_ = 0.0129 S cm^−1^) and the nonaqueous 0.3 m TBABF_4_ in acetonitrile reference electrolyte (*σ*_ACN + 0.3 m TBABF4_ = 0.022 S cm^−1^).

	*R*_Ω_ [Ω] ISO7888 (H_2_O + 0.1 m KCl) + Daramic 175	*ζ* [cm^−1^] ISO7888 (H_2_O + 0.1 m KCl) + Daramic 175	*R*_Ω_ [Ω] Acetonitril + 0.3 m TBABF_4_ + Celgard 2500	*ζ* [cm^−1^] Acetonitril + 0.3 m TBABF_4_ + Celgard 2500
Coaxial Reference	372	4.8	218	4.8
−CF −S	11	0.14	8	0.18
−CF +S	27	0.35	13	0.29
+CF +S	**13**	**0.17**	**2**	**0.04**

As the electrode surface area increases when switching from the stainless steel coaxial reference cell to the bare 3D‐printed lab‐scale RFB cell without separator and carbon felt (−CF‐S) with only the graphite blocks as current collectors, the bulk resistance and correspondingly the cell constant also decrease. By adding a separator (−CF+S, for aqueous electrolyte Daramic 175 and for nonaqueous Celgard 2500), an additional resistance is inserted between the two graphite blocks, which increases *R*_Ω_ and *ζ*. With a further increase in electrode surface area and decrease in interelectrode distance by adding carbon felt, *R*_Ω_ drops again (in accordance with the bulk resistance from Section 3., *R*_Ω, Fc|FcBF4_ = 2 Ω) and thus so does ζ, resulting in a ζ_H2O_, _+CF+S_ = 13 cm^−1^ and a ζ_ACN_, _+CF+S_ = 2 cm^−1^ for the complete 3D‐printed lab‐scale RFB structure (as appliead to the charge–discharge experiments in Section 5). These overall experimentally achieved ζ for the 3D‐printed lab‐scale RFB fit also in the theoretically expected range for the RFB cell constant calculated via the RFB setup dimensions using Equation (7): ζ_theo_ = d / A = 0.5 cm / 1.92 cm^2^ = 0.26 cm^−1^. The difference between the aqueous and nonaqueous calibration electrolyte resistances can presumably be attributed to the different types of conducting salt and separators. Despite the still subtle differences, this stepwise increase in complexity paints a comprehensive picture of the impeding elements in an RFB setup and this procedure thus ensures the user that the value of each component fits within a reasonable margin.

## 8 Identifying the RFB Setup Independent Minimum Operating Parameter Ratio *K*2

Since the cell constant is describing quantitatively the cell geometry, it can be used, just like in classical conductivity measurements (*ζ*), first to quantitatively compare the influence of the individual RFB cell structure on the conductivity and, in extension, the cycling performance. Second, it can be applied to convert *K*1_critical_ into efficiency coefficients for a specific RFB cell structure independent of this cell structure influence (*K*2). Therefore, *K*2 is introduced here as the cell structure independent value auf *K*1_critical_ and is calculated according to Equation (10)

(10) $$\begin{array}{c} K2_{\mathrm{non}-\mathrm{aq}.}=\frac{K1_{\mathrm{critical}}}{\zeta }=\frac{55}{2\;\;cm^{-1}}=28\;\;\mathrm{cm}\\ K2_{\mathrm{aq}.}=\frac{K1_{\mathrm{critical}}}{\zeta }=\frac{66}{2\;\;cm^{-1}}=33\;\;\mathrm{cm} \end{array}$$

*K*2 and *ζ* can be used to compare the *K*1_critical_ obtained in different cell design and electrolyte chemistries as well as their certain efficiencies in different operating conditions described by *K*1. The procedure for determining *K*1 and *K*1_critical_ presented in the first section of this paper thus becomes independent of the RFB cell geometry and different RFB designs can be compared and evaluated in their optimum efficiency ranges (defined by *K*1 and *K*1_critical_). This hopefully makes it easier to compare individual RFB performance results using different RAS, electrolyte combinations, separators, electrodes, flow fields, etc.

Furthermore, it serves as a first benchmark to identify the area for *K*1_critical_ using the measured *ζ* for a certain RFB cell setup, when using a new, unknown RFB setup and evaluating the ideal performance and efficiency window.

## 9 How to Use *K*1, *K*1_critical_, *ζ*, and *K*2?

To identify the individual lab‐scale RFB parameters *K*1_critical_, *ζ*, and *K*2 using the molar flux balancing mass‐transport perspective and the corresponding experimental route on efficiency evaluation presented in this work, these instructions can be followed

1. Identify the RFB cell constant *ζ* according to ISO 7888 for aqueous systems or the 0.3 m TBABF_4_‐acetonitrile reference supporting electrolyte for nonaqueous systems, measuring the bulk resistance via EIS and calculating *ζ* via Equation (7) while gradually increasing the RFB systems complexity.
2. Use the herein measured *K*2 ≈ 28 cm for nonaqueous and *K*2 ≈ 33 cm for aqueous electrolyte as first benchmark to estimate *K*1_critical_ via Equation (8).
3. Set up a *K*1 matrix (minimum 3 × 3 × 3) of different ${\dot{n}}_{p}$ and ${\dot{n}}_{c}$ (i.e., *c*_RAS_, $\dot{V}$, and *I*) around the as calculated *K*1_critical_ and screen the RFB cell performance via *NCE* and *η* evaluation at different *K*1 using Equations (4) and (5)
4. Identify the real *K*1_critical_ of the used setup at 110% of *η*_min_ from Equation (6).
5. Recalculate *K*2 using real *K*1_critical_ and *ζ* evaluated for the individual RFB setup.

Thus, *K*1_critical_, *ζ*, and *K*2 first provide optimal cycling parameters, i.e., the minimum ${\dot{n}}_{p}$ to ${\dot{n}}_{c}$ ratio for efficient charge–discharge cycling (*K*1_critical_), and second enable cross‐comparison of individual RFB experiments. However, in order to apply the experimental route presented here to RFB setups on a pilot plant scale or multistack systems, further investigations are required in the future, so that the standardized experimental route presented here for determining the optimum operating parameters and the basic comparability of different RFB setups is first recommended for single‐stack, lab‐scale RFB setups for the time being.

## 10 Conclusion and Outlook

By relating electrochemical parameters such as applied current, resistance, and overvoltage of a lab‐scale RFB with parameters relevant to mass transport, comprised of a productive molar flux (${\dot{n}}_{p}\;/\;\mathrm{mol}\;s^{-1}$) and consumptive molar flux (${\dot{n}}_{c}/\mathrm{mol}\;s^{-1}$), we show that one can directly identify the effect of different operating parameters (i.e., RAS concentration, electrolyte flow rate, and applied current) on lab‐scale RFB performance with a *K*1 parameter, thereby simplifying RFB optimization. To determine optimal settings, a simple experimental calibration route that monitors the change in RFB overvoltage (*η*/V) and normalized charge efficiency (*NCE*/%) upon varying the ratio of productive to consumptive molar flux (i.e., varying *K*1 values) is presented, using aqueous (K_4_[Fe(CN)_6_]|K_3_[Fe(CN)_6_], 0.1 m KCl, deionised water) and nonaqueous (Fc|FcBF_4_, 0.3 m TBABF_4_, acetonitrile) standard calibration electrolytes. The critical *K*1 ratio of the productive and consumptive molar fluxes that yields efficient charge–discharge cycling is achieved by having sufficient RAS mass‐transport inside the individual RFB setup. *K*1_critical_ thus serves as a setup specific parameter, benchmarking charge–discharge efficiency for different lab‐scale RFB setups. Although it generally would be desirable to increase the flow rate during cycling to counteract the decrease in unreacted RAS and to thus preserve optimized cycling conditions also at high SoC, we have fixed *K*1_critical_ as a static value for RFB cycling based on our experimental and literature values at 10% over minimum overvoltage. Here, we demonstrate our experimental route using an earlier published lab‐scale RFB, amounting a *K*1_critical, nonaqueous_ *=* 55 and *K*1_critical , aqueous_ *=* 66 (both for porous separators), thus identifying a need for a large excess of productive against consumptive molar flux for critical efficient operating parameters. Importantly, these values are in the range of identified literature median values of ${\tilde{K1}}_{\mathrm{porous}}$ = 43 for porous separators and ${\tilde{K1}}_{\mathrm{IEM}}$ = 96 for ion exchange membranes, validating the assessment. Furthermore, through implementation of the RFB cell constant *ζ*, an optimum efficiency range for cycling procedures can be determined irrespective of RFB cell geometry using *K*2, thus enabling cross‐comparison between RFB setups from different studies. Ultimately, this straightforward mass transport approach offers a simple experimental route to enable comparison of experimental results between different RFB cell setups, offering a unique opportunity to optimize the electrochemical parameters of lab‐scale RFBs in general.

## 11 Experimental Section

The lab‐scale RFB and pump setup used was the previously reported 3D‐printed lab‐scale RFB setup (electrochemical active area 2.4 cm^2^). SIGRACELL GFD 2.5 EA IW1 cut to size was used as carbon felt.

For the aqueous electrolyte, 25, 50, and 75 mm of K_3_[Fe^III^(CN)_6_] or K_4_[Fe^II^(CN)_6_] • 3 H_2_O were dissolved in Milli‐Q water together with 0.1 m KCl as supporting electrolyte. 3 mL of each solution was added as anolyte and catholyte electrolyte into the Eppendorf Tubes that served as the tanks, resulting in theoretical maximum capacities of 4.02, 8.04, and 12.06 mAh. The aqueous electrolyte was galvanostatically cycled at 20, 30, 40, and 60 mA between +0.7 and −0.7 cut off voltage and a volume flow rate of 5, 10, or 15 mL min^−1^. Aqueous electrolyte experiments were performed under ambient conditions using a Daramic 175 separator.

The nonaqueous electrolyte formulation proposed by Armstrong et al. based on ferrocene (Fc) and ferrocenium tetrafluoroborate (FcBF_4_) was used as the standard calibration electrolyte. For this purpose, 2.5, 5, and 7.5 mm Fc and FcBF_4_ were dissolved in acetonitrile (dried on molecular sieves in an argon‐filled glovebox) and 0.3 m tetrabutylammonium tetrafluoroborate (TBABF_4_) was added as the conducting salt. The TBABF_4_ was previously dried at 120 °C and under vacuum. 3 mL of this electrolyte were filled into Eppendorf Tubes, which served as the tanks, and connected to the 3D‐printed lab‐scale RFB setup. Nonaqueoues electrolytes were performed in a nitrogen‐filled glovebag, that was flushed with nitrogen before every measurement to ensure H_2_O and O_2_ exclusion (Figure S1, Supporting Information), using a Celgard 2500 separator.

The electrolyte was pumped with 5, 10, 20, and 30 mL min^−1^ which was determined by a previous gravimetric pump calibration.

It should be pointed out that both symmetrical standard calibration [Fe^II^(CN)_6_]^3−^|[Fe^III^(CN)_6_]^4−^ Fc|FcBF_4_ electrolyte used here, just like for reference calibration in cyclic voltammetry measurements, only serves as a tool for characterizing an individual lab‐scale RFB setup. Using these standardized electrolytes, the experimental route proposed here (based on the variation between productive and consumptive molar flux accompanied by the *NCE* and overvoltage efficiency evaluation) can be used to determine the RFB setup‐specific influence on the charge–discharge cycling behavior. Finally, this gets condensed in a setup‐specific, individual *K*1_critical_, the minimal *K*1 ratio required to achieve sufficient cycling efficiency. This standardized experimental route then can be used to compare the cycling quality of separate RFB setups with each other. Providing the here presented *K*1_critical_, *ζ*, and *K*2 values upcoming research articles would facilitate cross‐comparison of results between different RFB designs.

For the evaluation of the cell constant, a conductance standard according to ISO 7888 0.1 m KCl solution was used supplied by Sigma‐Aldrich.

An Autolab multipotentiostat from Metrohm PGSTAT 204N with Nova 2.1 software was used for the electrochemical investigations. The used RFB cell and pump setup was the previously reported 3D‐printed RFB setup, which was stored and processed in a nitrogen‐filled glovebag.^[^ ^28^ ^]^ Cycling performance was evaluated with 5/−5, 10/−10, or 15/−15 mA for nonaqueous and at 20/−20, 30/−30, 40/−40, and 60/−60 mA for aqueous electrolyte. Cut‐off polarisation potentials were set at 1 and −1 V for nonaqueous and at 0.7 and −0.7 V for aqueous electrolyte experiments. Four charge discharge cycles were measured per measuring point. The average values of the second, third, and fourth cycle were used for the quantitative evaluation and the second cycle was used as displayed in Figure 5; and Figures S3–S5 (Supporting Information) for nonaqueous and Figures S7–S9 (Supporting Information) for aqueous electrolyte. The efficiencies were calculated according to Equations (11) and (12) and ((13), (14), (15))

(11) $$NCE=\left(Q_{c.\;\exp.}/Q_{c.\mathrm{theo}.}\right)\cdot 100$$

(12) $$\eta =\left(\left|{\bar{U}}_{\mathrm{discharge}}\right|+{\bar{U}}_{\mathrm{charge}}\right)/2$$

(13) $$CE=t_{\mathrm{discharge}}/t_{\mathrm{charge}}$$

(14) $$VE={\bar{U}}_{\mathrm{discharge}}/{\bar{U}}_{\mathrm{charge}}$$

(15) EE=CE·VE

It should be noted at this point that the typical electrochemical evaluation parameters (Coulomb efficiency (*CE*), voltage efficiency (*VE*), and energy efficiency (*EE*)) have only limited significance here, since

1. Due to the electrochemical stability and fast and reversible redox kinetics and reaction of the symmetrical RAS species Fe^II+^|Fe^III+^ used here, the evaluation of the galvanostatic cycling results obtained can be attributed only to the mass transport phenomena presented in this work and other electrochemical difficulties (e.g., by low electrochemical stability, slow redox kinetics.) can be neglected. Furthermore, since the reversibility of the redox reaction within a charge–discharge cycle is of less interest than the charging capacity achieved relative (*Q*_c. exp._) to the theoretically possible (*Q*_c. theo._) in order to determine the electrochemically converted amount of RAS for the respective operating parameters (*K*1), the normalized charge efficiency (*NCE*), and not the *CE* is used for performance evaluation in this work.
2. The theoretical cell voltage of the symmetric Fe^II+^|Fe^III+^ electrolyte between the anodically polarized Fc and the cathodically polarized Fc^+^ should be zero and only the polarization should change from Fe^II+^|Fe^III+^ to Fe^III+^|Fe^II+^ and vice versa during the respective “charge” and “discharge” steps. Thus, the *VE* is of less interest for the present investigation, but only the overvoltage (*η*) induced by mass transport limitations is used as a performance criterion. Since the overvoltage is the difference between the theoretical voltage and the real nominal voltage, whereby the nominal voltage for the symmetrical electrolyte used here is 0 V, any deviation in the cell voltage is due to overvoltage and can be calculated here by Equation (5)). For actual RFB systems, which build up an “real” nominal voltage between anolyte and catholyte, the overvoltage could be determined by running a symmetric cell of the involved electrolyte instead. However, this will not safeguard the stability of the RFB system during the determination of overvoltage (i.e., cross‐over and side/decomposition reactions can occur), which is why the use of the Fe^II+^|Fe^III+^ electrolyte still recommended for calibration.
3. Since the *CE* and *VE* have less importance as described above, also *EE* is not used for the evaluation of the energy efficiency.

However, for completeness, all three variables have been included in the corresponding investigations (Tables S3–S5, (Supporting Information)).

Impedance spectra were measured in a frequency range from 0.01 to 10^6^ Hz with an amplitude of 0.01 *V*_RMS_. Impedance spectroscopy results were evaluated using the RelaxIS software. A detailed evaluation of the influence of ${\dot{n}}_{p}$ on EIS experiments and of the cell constant experimental route can be found in the Supporting Information.

## Conflict of Interest

The authors declare no conflict of interest.

## Supporting information



Supporting Information

## Data Availability

The data that support the findings of this study are available from the corresponding author upon reasonable request.
