# Supplementary material for: Introducing an Experimental Route to Identify and Unify Lab‐Scale Redox‐Flow Battery Cell Performances via Molar Fluxes and Cell Constants
Source: Small Methods. 2025 May 28;9(7):2401670. doi: 10.1002/smtd.202401670 (PMC12285640; doi:10.1002/smtd.202401670)
Supplement: Supplementary file 1 — Supporting Information [file SMTD-9-2401670-s001.docx]

Supporting Information

**Introducing an Experimental Route to Identify and Unify Lab-Scale Redox-Flow Battery Cell Performances v*ia* Molar Fluxes and Cell Constants**

Sebastian Fricke, Luuk Kortekaas, Martin Winter and Mariano Grünebaum*

S. Fricke, M. Winter, M. Grünebaum

Helmholtz-Institute Münster, IMD-4, Forschungszentrum Jülich GmbH, Corrensstraße 48, 48149 Münster, Germany

E-mail: m.gruenebaum@fz-juelich.de

L. Kortekaas

Materials Chemistry, Faculty of Science and Engineering, University of Groningen, 9747 AG

Groningen, The Netherlands

M. Winter

MEET Battery Research Center, University of Münster, Corrensstraße 46, 48149 Münster, Germany

List of all RFB operating and performance metrics for RFB cell performance evaluation via molar fluxes

**Table S 1**. Summary of all used abbreviations, their units, calculation and explanation.

| Abbreviation | Calculation | Unit | Note |
| --- | --- | --- | --- |
| *c_RAS_* | (given) | mol l^-1^ | Concentration of redox-active species (RAS). |
| *z* | (given) | - | Number of electrons transferred (for the Fc\|FcBF_4_ standard calibration electrolyte z = 1). |
| $\dot{\boldsymbol{V}}$ | (given) | mL min^-1^ | Volume flow rate of the electrolyte through the RFB cell. |
| *I* | (given) | mA | Applied charge and discharge current during cycling. |
| *F* | $F=96485$ | A s mol^-1^ | Faraday constant. |
| $\dot{\boldsymbol{n}_{\boldsymbol{p}}}$ | $\dot{n_{p}}={z\cdot c}_{RAS}\cdot\dot{V}$ | mol s^-1^ | Productive molar flux. |
| $\dot{\boldsymbol{n}_{\boldsymbol{c}}}$ | $\dot{n_{c}}=I/{(z\cdot F)}$ | mol s^-1^ | Consumptive molar flux. |
| *K1* | $K1={\dot{n_{p}}}/{\dot{n_{c}}}$ | - | Ratio between productive and consumptive molar flux, used for efficiency evaluation. |
| *K1_critical_* | ${K1}_{critical}= K1\left( at 110\% \eta_{min} \right)$ | - | Ratio between productive and consumptive molar flux, to run the cell at a 110% of minimum overvoltage. |
| *K1* >> *K1_critical_* | *-* | - | Limiting Case 1: $\dot{n_{p}}$ to the electrode far exceeds the demand by $\dot{n_{c}}$. |
| *K1* << *K1_critical_* | *-* | - | Limiting Case 2: Consumptive $\dot{n_{c}}$ exceeds the available productive $\dot{n_{p}}$. |
| *ζ* | $\zeta={spec. cond.}/{abs. cond.}= \sigma/\Sigma=\sigma\cdot R_{\Omega}$ | cm^-1^ | RFB cell constant, experimentally calculated *via* given specific conductivity, $\sigma$, of standard calibration electrolytes ISO7888 for aqueous systems and 0.3 M TBABF_4_ in acetonitrile for non-aqueous ones, and measured bulk-resistance, $R_{\Omega}$ ($R_{\Omega}=\frac{1}{\Sigma}$), via EIS, to overall create quantitative comparability between different RFB setup. |
| *K2* | $K2={{K1}_{critical}}/\zeta$ | cm | Setup-independent ${K1}_{critical}$, allowing comparability between different RFB setups and their charge efficiency. |
| *η (η_min_)* | $\eta={\left\vert{(\bar{U}}_{d} \right\vert+\bar{U}_{c})}/2$ | V | (Minimum) Overvoltage for a symmetric RFB setup. |
| *NCE* | $NCE={t_{c, measured}}/{t_{c, theoretical}}$ | % | Normalized charge efficiency, on how much RAS has been electrochemically converted relative to the theoretically possible amount. |

Installation picture of the lab-scale RFB setup used

A previously published 3D-printed RFB design (electrochemical active area 2.4 cm²) for aqueous and nonaqueous electrolytes was used. [28] The RFB setup was used in a nitrogen-flooded glovebag under constant overpressure, ensuring a consistent nitrogen atmosphere inside the glovebag. A more detailed explanation on the setup used can be found in the corresponding reference.


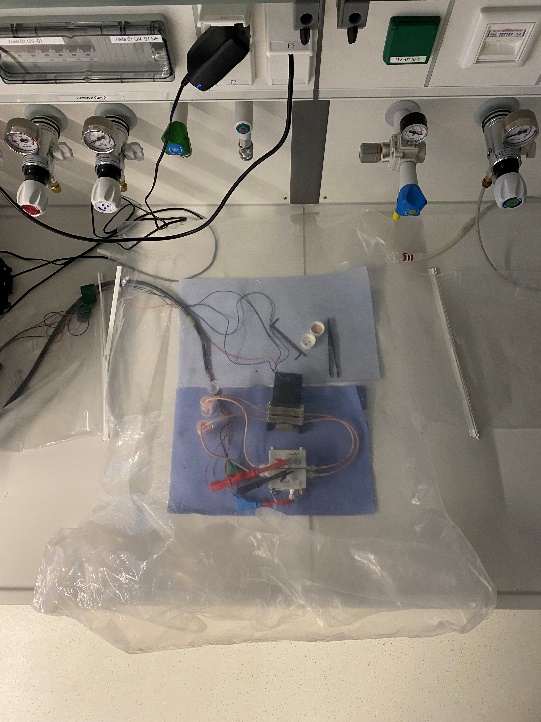


**Figure S 1.** 3D-printed lab-scale RFB setup in a nitrogen flooded glove bag.

**Impact of** $\dot{\boldsymbol{n}_{\boldsymbol{p}}}$ **on conductivities within a lab-scale RFB cell**

In order to test the influence of productive molar flux $\dot{n_{P}}$ on the resistances of the individual processes within a flow battery, the Fc|FcBF_4_, non-aqueous, standard calibration electrolyte (with acetonitrile and 0.3 M TBABF_4_ supporting electrolyte) was pumped through the here used reference setup (2.4 cm², flow-through parallel cell setup; more details can be found in [28]) at three different RAS concentrations (*c_RAS_* = 5, 10 and 15 mM) at four different flow rates ($\dot{V}$ = 5, 10, 20, 30 mL min^-1^) and electrochemical impedance spectroscopy were performed at frequency range from 0.1 Hz to 10^6^ Hz with an amplitude of 0.01 *V_RMS_*. For evaluation of R_Ω_, the high-frequency x-axis intercept was simply read off in the RelaxIS^©^ evaluation software. For the evaluation of the raw data (**Figure 3 b)** and **Figure S2 a)** and **b)** dots) of *R_CT_* and *R_MT_*, the middle- and high-frequency two right half-circle x-axis intercepts were individually fitted with RelaxIS (Figure 3 b) and Figure S2 a) and b) continuous line). The low-frequency right half-circle x-axis intercept was extrapolated afterwards (Figure 3 b) and Figure S2 a) and b) dashed lines), in accordance with the equivalent circuit in **Figure 3 a)**:

**Figure S 2.** EIS raw data evaluation (dots) including the fitted function (continuous line, 0.01 Hz to 1 MHz) and the simulation for lower frequencies (dashed line, 0.01 mHz to 1 MHz) of 0.3 M TBABF_4_ in acetonitrile supporting electrolyte with a) 5 mM Fc|FcBF_4_ and b) at flow rates between 5 and 30 mL min^-1^ using the equivalent circuits shown in Figure 3 a).

**Table S 2.** Overview of the bulk resistance (*R_Ω_* / Ω), charge-transfer resistance (*R_CT_* / Ω) and mass-transfer resistance (*R_CT_* / Ω) calculated from the EIS raw data (Figure 3 b) and Figure S2 a) and b) using the equivalent circuit (Figure 3 a)), according to RAS concentration (*c_RAS_* / mM), flow rate ($\dot{V}$ / mL min^-1^) and the resulting productive molar flux ($\dot{n_{p}}$/ mmol s^-1^). Graphically evaluated in Figure 4).

| *c_RAS_* / mM | $\dot{\boldsymbol{V}}$ / mL min^-1^ | $\dot{\boldsymbol{n}_{\boldsymbol{p}}}$/ mmol s^-1^ | *R_Ω_* / Ω | *R_CT_*/ Ω | *R_MT_* / Ω |
| --- | --- | --- | --- | --- | --- |
| 15 mM | 5 | 0.00125 | 2.4 | 2.6 | 18.7 |
|  | 10 | 0.0025 | 2.3 | 2.6 | 6.2 |
|  | 20 | 0.005 | 2.4 | 2.9 | 3.5 |
|  | 30 | 0.0075 | 2.4 | 3.23 | 2.5 |
| 10 mM | 5 | 8.33333E-4 | 2.3 | 6.3 | 17.1 |
|  | 10 | 0.00167 | 2.3 | 6.3 | 10.1 |
|  | 20 | 0.00333 | 2.3 | 2.4 | 6.0 |
|  | 30 | 0.005 | 2.4 | 3.4 | 2.9 |
| 5 mM | 5 | 4.16667E-4 | 2.1 | 3.8 | 42.8 |
|  | 10 | 8.33333E-4 | 2.2 | 2.6 | 32.2 |
|  | 20 | 0.00167 | 2.1 | 4.0 | 6.1 |
|  | 30 | 0.0025 | 2.1 | 4.1 | 4.0 |

Polarisation curves and their evaluation of non-aqueous charge-discharge experiments containing 5, 10 and 15 mM Fc|FcBF_4_ RAS cycled at 5, 10 and 15 mA applied current with 5, 10 20 and 30 mL min^-1^ flow rate

**Figure S 3**. Polarization curves of cells containing 5 mM Fc|FcBF4 electrolyte at (A) 5 mA, (B) 10 mA and (C) 15 mA.

**Figure S 4.** Polarization curves of cells containing 10 mM Fc|FcBF4 electrolyte at (A) 5 mA, (B) 10 mA and (C) 15 mA.

**Figure S 5.** Polarization curves of cells containing 15 mM Fc|FcBF4 electrolyte at (A) 5 mA, (B) 10 mA and (C) 15 mA.

Summary of the overall basic efficiencies (Coulombic efficiency (*CE* / %), voltage efficiency (*VE* / %), energy efficiency (*EE* / %)) the ones for the symmetric non-aqueous Fc|FcBF_4_ calibration electrolyte more important ones (*NCE* and *η*) of the charge-discharge cycling polarization curves shown in Figure 4. The values are calculated as shown in the experimental section using Equation 8-12:

**Table S 3.** Efficiencies of cells containing different Fc|FcBF4 electrolyte concentrations at constant flow rate of 20 mL min^-1^ and 5 mA current (Figure 4a):

| Concentration (*c_RAS_*) | *CE* / % | | *VE* / % | *EE* / % | *NCE* / % | *η* / V |
| --- | --- | --- | --- | --- | --- | --- |
| 5 mM | 94.33 | 95.46 | | 90.04 | 52.74 | 0.21 |
| 10 mM | 91.93 | 83.33 | | 76.61 | 88.49 | 0.11 |
| 15 mM | 92.64 | 90.00 | | 83.38 | 91.03 | 0.10 |

**Table S 4.** Efficiencies determined considering different flow rates at constant RAS concentration of 5 mM and 5 mA current (Figure 4b):

| Flow rate ($\dot{\boldsymbol{V}}$) | *CE* / % | *VE* / % | *EE* / % | *NCE* / % | *η* / V |
| --- | --- | --- | --- | --- | --- |
| 30 mL min^-1^ | 92.64 | 90.00 | 83.48 | 61.03 | 0.19 |
| 20 mL min^-1^ | 94.33 | 95.46 | 90.04 | 52.74 | 0.22 |
| 10 mL min^-1^ | 75.84 | 92.86 | 70.42 | 9.44 | 0.41 |
| 5 mL min^-1^ | 89.47 | 1.09 | 97.14 | 6.56 | 0.39 |

**Table S 5.** Efficiencies determined considering different applied currents at constant RAS concentrations of 5 mM and volume flow rates at 20 mL min^-1^ (Figure 4c):

| Current (*I*) | *CE* / % | *VE* / % | *EE* / % | *NCE* / % | *η* / V |  |
| --- | --- | --- | --- | --- | --- | --- |
| 5 mA | 94.33 | 95.46 | 90.04 | 52.74 | 0.21 | |
| 10 mA | 67.38 | 100.00 | 67.38 | 14.94 | 0.49 | |
| 15 mA | 100.00 | 1.22 | 1.22 | 7.90 | 0.92 | |

Comparison of polarization curves originating from different operating parameters combination of the same *K1* value for non-aqueous Fc|FcBF_4_ electrolyte. *K1*s compared are 32 and 48.


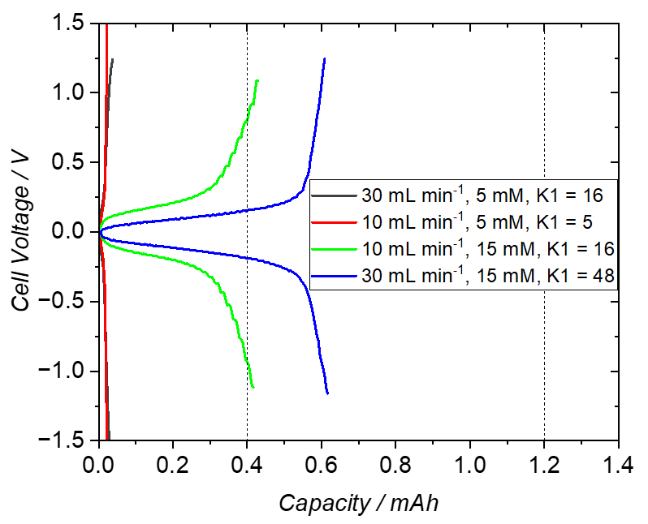


**Figure S 6.** Influence of volume flow rate (10 mL min^-1^ and 30 mL min^-1^) versus concentration (5 mM and 15 mM) during cycling of cells containing the Fc|FcBF_4_ standard at a constant current (15 mA; *K1* = 5, 16, 16 and 48). The dashed lines show the theoretical possible capacity for 5 mM RAS (0.4 mAh) and 15 mM (1.2 mAh).

**Table S 6**. Comparison of same *K1* = 32 and 48 of non-aqueous Fc|FcBF4 performance evaluation composed by different $c_{RAS}$, $\dot{V}$ and *I* and their corresponding *NCE*:

| $\boldsymbol{K}\boldsymbol{1}$ | $\boldsymbol{c}_{\boldsymbol{RAS}}$ / mM | $\dot{\boldsymbol{V}}$ / mL min-1 | $\boldsymbol{I}$ / mA | $\boldsymbol{NCE}$ / % | *η* / V |
| --- | --- | --- | --- | --- | --- |
| 32 | 5 | 20 | 5 | 53 | 0.22 |
|  | 10 | 10 | 5 | 69 | 0.17 |
|  | 10 | 20 | 10 | 68 | 0.18 |
|  | 10 | 30 | 15 | 61 | 0.25 |
|  | 15 | 20 | 15 | 20 | 0.24 |
|  |  |  |  |  |  |
| 48 | 5 | 30 | 5 | 61 | 0.19 |
|  | 10 | 30 | 10 | 69 | 0.16 |
|  | 15 | 10 | 5 | 72 | 0.15 |
|  | 15 | 30 | 15 | 58 | 0.11 |

**Table S 7.** Detailed overview of each applied *K1* and their corresponding overvoltage (*η*) and normalized charge efficiency (*NCE*) for non-aqueous performance evaluation with Fc|FcBF4 electrolyte.

| $\boldsymbol{c}_{\boldsymbol{RAS}}$ / mM | $\boldsymbol{I}$ / mA | $\dot{\boldsymbol{V}}$ / mL min^-1^ | *K1* | *η* / V | *NCE* / % |
| --- | --- | --- | --- | --- | --- |
| 5 | 5 | 5 | 8 | 0.36 | 6.56 |
|  |  | 10 | 16 | 0.39 | 9.44 |
|  |  | 20 | 32 | 0.21 | 52.74 |
|  |  | 30 | 48 | 0.19 | 61.03 |
|  | 10 | 5 | 4 | 0.77 | 6.66 |
|  |  | 10 | 8 | 0.67 | 6.89 |
|  |  | 20 | 16 | 0.49 | 14.94 |
|  |  | 30 | 24 | 0.46 | 29.45 |
|  | 15 | 5 | 2 | 1.04 | 7.21 |
|  |  | 10 | 5 | 1.05 | 7.21 |
|  |  | 20 | 10 | 0.92 | 7.90 |
|  |  | 30 | 16 | 0.73 | 9.62 |
| 10 | 5 | 5 | 16 | 0.36 | 42.08 |
|  |  | 10 | 32 | 0.16 | 69.60 |
|  |  | 20 | 64 | 0.11 | 88.48 |
|  |  | 30 | 96 | 0.10 | 83.36 |
|  | 10 | 5 | 8 | 0.42 | 11.30 |
|  |  | 10 | 16 | 0.35 | 42.90 |
|  |  | 20 | 32 | 0.18 | 68.16 |
|  |  | 30 | 48 | 0.15 | 69.66 |
|  | 15 | 5 | 5 | 0.48 | 7.42 |
|  |  | 10 | 10 | 0.49 | 16.75 |
|  |  | 20 | 21 | 0.28 | 53.02 |
|  |  | 30 | 32 | 0.25 | 60.79 |
| 15 | 5 | 5 | 24 | 0.25 | 54.49 |
|  |  | 10 | 48 | 0.15 | 72.35 |
|  |  | 20 | 96 | 0.10 | 91.01 |
|  |  | 30 | 144 | 0.12 | 83.64 |
|  | 10 | 5 | 12 | 0.40 | 11.52 |
|  |  | 10 | 24 | 0.29 | 40.55 |
|  |  | 20 | 48 | 0.16 | 70.27 |
|  |  | 30 | 72 | 0.14 | 67.28 |
|  | 15 | 5 | 8 | 0.41 | 6.92 |
|  |  | 10 | 16 | 0.31 | 13.14 |
|  |  | 20 | 32 | 0.23 | 19.26 |
|  |  | 30 | 48 | 0.19 | 58.47 |

Polarisation curves and their evaluation of aqueous charge-discharge experiments containing 50, 100 and 150 mM [Fe^III^(CN)_6_]^3-^|[Fe^II^(CN)_6_]^4-^ RAS cycled at 20, 30, 40 and 60 mA applied current with 5, 10 and 20 mL min^-1^ flow rate

**Figure S 7.** Polarization curves of cells containing 50 mM [Fe^III^(CN)_6_]^3-^|[Fe^II^(CN)_6_]^4-^ electrolyte at (A) 5 mL min^-1^, (B) 10 mL min^-1^ and (C) 20 mL min^-1^.

**Figure S 8.** Polarization curves of cells containing 100 mM [Fe^III^(CN)_6_]^3-^|[Fe^II^(CN)_6_]^4-^ electrolyte at (A) 5 mL min^-1^, (B) 10 mL min^-1^ and (C) 20 mL min^-1^.

**Figure S 9.** Polarization curves of cells containing 100 mM [Fe^III^(CN)_6_]^3-^|[Fe^II^(CN)_6_]^4-^ electrolyte at (A) 5 mL min^-1^, (B) 10 mL min^-1^ and (C) 20 mL min^-1^.

**Table S 8**. Detailed overview of each applied *K1* and their corresponding overvoltage (*η*) and normalized charge efficiency (*NCE*) for aqueous performance evaluation with [Fe^III^(CN)_6_]^3-^|[Fe^II^(CN)_6_]^4-^ electrolyte.

| $\boldsymbol{c}_{\boldsymbol{RAS}}$ / mM | $\dot{\boldsymbol{V}}$ / mL min^-1^ | $\boldsymbol{I}$ / mA | *K1* | *η* / V | *NCE* / % |
| --- | --- | --- | --- | --- | --- |
| 50 | 20 | 20 | 80 | 0.17 | 78 |
|  |  | 30 | 53 | 0.25 | 70 |
|  |  | 40 | 40 | 0.32 | 65 |
|  |  | 60 | 26 | 0.50 | 39 |
|  | 10 | 20 | 40 | 0.19 | 60 |
|  |  | 30 | 26 | 0.28 | 58 |
|  |  | 40 | 20 | 0.38 | 46 |
|  |  | 60 | 13 | 0.50 | 39 |
|  | 5 | 20 | 20 | 0.26 | 35 |
|  |  | 30 | 13 | 0.38 | 21 |
|  |  | 40 | 10 | 0.49 | 6 |
|  |  | 60 | 6 | 0.49 | 8 |
| 10 | 20 | 20 | 160 | 0.12 | 79 |
|  |  | 30 | 107 | 0.17 | 73 |
|  |  | 40 | 80 | 0.22 | 70 |
|  |  | 60 | 53 | 0.33 | 60 |
|  | 10 | 20 | 80 | 0.13 | 45 |
|  |  | 30 | 53 | 0.18 | 43 |
|  |  | 40 | 40 | 0.25 | 39 |
|  |  | 60 | 26 | 0.38 | 30 |
|  | 5 | 20 | 40 | 0.15 | 36 |
|  |  | 30 | 26 | 0.22 | 34 |
|  |  | 40 | 20 | 0.31 | 25 |
|  |  | 60 | 13 | 0.46 | 10 |
| 15 | 20 | 20 | 241 | 0.09 | 89 |
|  |  | 30 | 160 | 0.14 | 87 |
|  |  | 40 | 120 | 0.18 | 85 |
|  |  | 60 | 80 | 0.26 | 82 |
|  | 10 | 20 | 120 | 0.10 | 84 |
|  |  | 30 | 80 | 0.15 | 82 |
|  |  | 40 | 60 | 0.19 | 77 |
|  |  | 60 | 40 | 0.28 | 70 |
|  | 5 | 20 | 60 | 0.11 | 77 |
|  |  | 30 | 40 | 0.16 | 73 |
|  |  | 40 | 30 | 0.22 | 64 |
|  |  | 60 | 20 | 0.33 | 51 |

**Figure S 10.** (a) Overvoltage (*η* / V) and (b) normalized charge efficiency (*NCE* / %) plotted against different *K1* values applied for the aqueous [Fe^III^(CN)_6_]^3-^|[Fe^II^(CN)_6_]^4-^ electrolyte, here *η_min, aqueous_* = 0.14 V and *NCE_max, aqueoues_* = 82 % were achieved ($\eta_{{K1}_{critical, aq.}}$= 0.16 V) resulting in a *K1_critical, aq._* = 66.

Comparison of RFB literature cycling settings

**Table S 9.** Overview of experimental data obtained from literature on cycling (RAS concentration (*c_RAS_*), applied current (*I*) and volume flow rate ($\dot{V}$)) and their calculated productive and consumptive molar fluxes, $\dot{n_{p}}$plus $\dot{n_{c}}$, respectively, and the resulting *K1* ratios for porous separators and for ion exchange membranes. All compared systems were lab-scale size.

| Redox-active species | *c_RAS_* /  mol L^-1^ | Conducting  Salt | $\dot{\boldsymbol{V}}$ /  mL min^-1^ | *A /*  *cm²* | *j /*  *mA*  *cm^-2^* | *I* /  mA | $\dot{\boldsymbol{n}_{\boldsymbol{p}}}$/  mol s^-1^ | $\dot{\boldsymbol{n}_{\boldsymbol{c}}}$/  mol s^-1^ | *K1* | Source |
| --- | --- | --- | --- | --- | --- | --- | --- | --- | --- | --- |
| NBuPh-DMFc | 0.1 | TBABF_4_ | 10 | 4 | 60 | 240 | 1.67E-05 | 2.49E-06 | 6 | [38] |
| MePh-DBMMB | 0.3 | - | 50 | 20.1 | 35 | 703.5 | 2.50E-04 | 7.29E-06 | 34 | [39] |
| Fe(bpy)3(BF4)2/Fc1N112-BF4 | 0.1 | TBABF_4_ | 20 | 4.63 | 5 | 23.2 | 3.33E-05 | 2.40E-07 | 138 | [40] |
| AcNH-TEMPO | 0.05 | LIBF_4_ | 5 | 4.63 | 2 | 9.27 | 4.17E-06 | 9.61E-08 | 43 | [41] |
| V(acac)3 | 0.1 | TEABF_4_ | 25 | 5 | 10 | 50 | 4.17E-05 | 5.18E-07 | 80 | [42] |
| DBMMB-BzNSN | 0.1 | TEATFSI | 50 | 4 | 20 | 80 | 8.33E-05 | 8.29E-07 | 100 | [43] |
| DBMMB-BzNSN | 0.1 | LiTFSI | 20 | 5 | 40 | 200 | 3.33E-05 | 2.07E-06 | 16 | [44] |
| DBMMB-BzNSN | 0.5 | LiTFSI | 20 | 5 | 10 | 50 | 1.67E-04 | 5.18E-07 | 321 | [44] |
| BMEPZ/FL | 0.2 | LiTFSI | 80 | 4 | 20 | 80 | 2.67E-04 | 8.29E-07 | 321 | [45] |
| DMPZ-FL | 0.2 | LiTFSI | 80 | 4 | 20 | 80 | 2.67E-04 | 8.29E-07 | 321 | [46] |
| D (N-MEEEtBuPhePhtha-1) | 0.025 | TBAPF_6_ | 20 | 2.55 | 10 | 25.5 | 8.33E-06 | 2.64E-07 | 31 | [47] |
| 2-phenyl-4,4,5,5-tetramethylimidazoline-1-oxyl-3-oxide | 0.1 | TBAPF_6_ | 20 | 10 | 20 | 200 | 3.33E-05 | 2.07E-06 | 16 | [48] |
| 2-phenyl-4,4,5,5-tetramethylimidazoline-1-oxyl-3-oxide | 0.5 | TBAPF_6_ | 20 | 10 | 20 | 200 | 1.67E-04 | 2.07E-06 | 80 | [48] |
| Fe(BiPy)3(BF4)2-Ni(BiPy)3(BF4)2 | 0.4 | TEABF_4_ | 2.5 | 5 | - | 10 | 1.67E-05 | 1.04E-07 | 160 | [49] |
| V(acac)3 | 0.1 | TEABF_4_ | 25 | 5 | 10 | 50 | 4.17E-05 | 5.18E-07 | 80 | [42] |
| [Fe(phen)3](PF6)2-[Co(phen)3](PF6)2 | 0.01 | TEAPF_6_ | 20 | 36 | 0.13 | 4.68 | 3.33E-06 | 4.85E-08 | 68 | [50] |
| [Fe(phen)3](PF6)2-[Co(phen)3](PF6)2 | 0.01 | TEAPF_6_ | 20 | 36 | 0.25 | 9 | 3.33E-06 | 9.33E-08 | 35 | [50] |
| BP-TEMPO | 0.003 | TEAPF_6_ | 25 | 36 | 0.5 | 18 | 1.25E-06 | 1.87E-07 | 6 | [51] |
| Nitroxide Radical | 0.1 | TBAPF_6_ | 10 | 5 | 1 | 5 | 1.67E-05 | 5.18E-08 | 321 | [52] |
| Nitroxide Radical | 0.2 | TBAPF_6_ | 10 | 5 | 1 | 5 | 3.33E-05 | 5.18E-08 | 643 | [52] |
| FcNCl/MV | 0.5 | NaCl | 60 | 10 | 60 | 600 | 5.00E-04 | 6.22E-06 | 80 | [53] |
| BTMAP-Vi/BTMAP-Fc pH 7 | 1.3 | NaCl | 60 | 5 | 50 | 250 | 0.0013 | 2.59E-06 | 501 | [54] |
| DHDMBS/AQDS | 1.0 | H_2_SO_4_ | 150 | 25 | 100 | 2500 | 0.0025 | 2.59E-05 | 96 | [55] |
| ACA Ferrocyanide | 0.5 | KOH | 60 | 5 | 100 | 500 | 5.00E-04 | 5.18E-06 | 96 | [56] |
| TEMPTMA/MV | 2.0 | NaCl | 20 | 5 | 80 | 400 | 6.67E-04 | 4.15E-06 | 160 | [57] |

Evaluation of the cell constant *ζ*

To determine the RFB cell constant, *ζ* (/ cm^-1^), of a lab-scale RFB cell, a stepwise EIS experimental route with different complexity levels using a reference aqueous (ISO 7888 0.1 M KCl in water with a Daramic^©^ 175 porous separator) and non-aqueous (0.3 M TBABF4 in acetonitrile with a Celgard^©^ 2500 porous separator) electrolyte was performed on the aforementioned 3D-printed lab-scale RFB at no volume flow conditions. [28] It should be noted that a Celgard^©^ separator could not be used for the aqueous electrolyte because its highly hydrophobic nature greatly impedes contact between the electrodes, which is why the porous Daramic^©^ 175 separator was used for this purpose. To determine ζ, EIS measurements were performed in the frequency range from 1 MHz to 0.1 Hz and to calculate it, only the bulk resistance (*R*_Ω_ / Ω; first x-axis intercept) was read from the measured EIS spectra **(Figure S 11 a) – c)**). For a base cell constant, no RAS electron transfer kinetics are involved (as no RAS is included in the calibration electrolyte), thus the ohmic resistance is the relevant parameter for the cell constant evaluation. The following equation was used to calculate ***ζ*** using the absolute conductivity (*σ*_electrolyte_ / (S cm^-1^)) of the reference electrolytes:

|  | ***ζ* = *R*_Ω_ ^•^ *σ*_electrolyte_** |  |
| --- | --- | --- |

In a first step, the specific conductivity of the non-aqueous reference electrolyte was calculated (Figure S 11 a)) by determining the cell constant of the coaxial reference cell using the specific conductivity of the ISO 7888 standard (*σ*_ISO7888_ = 0.0129 S cm^-1^):

|  | ***ζ*_coax.ref._** = 372 Ω • 0.0129 S cm^-1^ = 4.8 cm^-1^  ***σ*_ACN + 0.3 M TBABF4_** = 4.8 cm^-1^ / 218 Ω = 0.022 S cm^-1^ |  |
| --- | --- | --- |

Subsequently, the cell constants of the respective cell setup stages were determined using these specific conductivities (Figure S 11 b) and Figure S 11 c): 1) bare graphite blocks in RFB setup without carbon felt and separator (-CF-S), 2) RFB setup without carbon felt but with separator (-CF+S) and 3) complete RFB setup including carbon felt and separator (+CF+S). As a zero-gap setup was used, it was not possible to carry out a setup stage with carbon felt but without separator, as otherwise there would be a short circuit between the electrodes. All measured impedance spectra and bulk resistances as well as the corresponding cell constants can be found in Table 1. In general, although it is not strictly necessary to go through each individual complexity step to determine only the bulk resistance of the final cell, we recommend this sequence to the reader for ensuring that the different cell component influences fall within an expected impedance range.

**Figure S 11**. EIS raw data of a) coaxial references with both ISO 7888 and non-aqueous 0.3 M TBABF_4_ in acetonitrile reference electrolyte, b) 3D-printed lab-scale RFB setup with ISO 7888 reference electrolyte and c) 3D-printed lab-scale RFB setup with non-aqueous 0.3 M TBABF_4_ in acetonitrile reference electrolyte.

References

1 Alotto, P., Guarnieri, M., Moro, F. (2014) Redox flow batteries for the storage of renewable energy: A review. *Renewable and Sustainable Energy Reviews*, **29**, 325–335.

2 Weber, A.Z., Mench, M.M., Meyers, J.P., Ross, P.N., Gostick, J.T., Liu, Q. (2011) Redox flow batteries: a review. *J Appl Electrochem*, **41** (10), 1137–1164.

3 Winsberg, J., Hagemann, T., Janoschka, T., Hager, M.D., Schubert, U.S. (2017) Redox-Flow Batteries: From Metals to Organic Redox-Active Materials. *Angewandte Chemie (International ed. in English)*, **56** (3), 686–711.

4 Park, M., Ryu, J., Wang, W., Cho, J. (2017) Material design and engineering of next-generation flow-battery technologies. *Nat Rev Mater*, **2** (1).

5 Wei, X., Pan, W., Duan, W., Hollas, A., Yang, Z., Li, B., Nie, Z., Liu, J., Reed, D., Wang, W., Sprenkle, V. (2017) Materials and Systems for Organic Redox Flow Batteries: Status and Challenges. *ACS Energy Lett.*, **2** (9), 2187–2204.

6 Gong, K., Fang, Q., Gu, S., Li, S.F.Y., Yan, Y. (2015) Nonaqueous redox-flow batteries: organic solvents, supporting electrolytes, and redox pairs. *Energy Environ. Sci.*, **8** (12), 3515–3530.

7 Cao, L., Skyllas-Kazacos, M., Menictas, C., Noack, J. (2018) A review of electrolyte additives and impurities in vanadium redox flow batteries. *Journal of Energy Chemistry*, **27** (5), 1269–1291.

8 Machado, C.A., Brown, G.O., Yang, R., Hopkins, T.E., Pribyl, J.G., Epps, T.H. (2021) Redox Flow Battery Membranes: Improving Battery Performance by Leveraging Structure–Property Relationships. *ACS Energy Lett.*, **6** (1), 158–176.

9 Yuan, J., Pan, Z.-Z., Jin, Y., Qiu, Q., Zhang, C., Zhao, Y., Li, Y. (2021) Membranes in non-aqueous redox flow battery: A review. *Journal of Power Sources*, **500**, 229983.

10 Tsehaye, M.T., Mourouga, G., Schmidt, T.J., Schumacher, J.O., Velizarov, S., van der Bruggen, B., Alloin, F., Iojoiu, C. (2023) Towards optimized membranes for aqueous organic redox flow batteries: Correlation between membrane properties and cell performance. *Renewable and Sustainable Energy Reviews*, **173**, 113059.

11 Kortekaas, L., Fricke, S., Korshunov, A., Cekic-Laskovic, I., Winter, M., Grünebaum, M. (2023) Building Bridges: Unifying Design and Development Aspects for Advancing Non-Aqueous Redox-Flow Batteries. *Batteries*, **9** (1), 4.

12 Huang, Z., Mu, A., Wu, L., Wang, H. (2022) Vanadium redox flow batteries: Flow field design and flow rate optimization. *Journal of Energy Storage*, **45**, 103526.

13 Yao, Y., Lei, J., Shi, Y., Ai, F., Lu, Y.-C. (2021) Assessment methods and performance metrics for redox flow batteries. *Nat Energy*, **6** (6), 582–588.

14 Choi, Y.Y., Oh, G., Choi, J.-I., Kim, Y., Kim, K.J. (2018) Pre-design model for redox flow battery design. *J Mech Sci Technol*, **32** (3), 1025–1032.

15 Aramendia, I., Fernandez-Gamiz, U., Martinez-San-Vicente, A., Zulueta, E., Lopez-Guede, J.M. (2021) Vanadium Redox Flow Batteries: A Review Oriented to Fluid-Dynamic Optimization. *Energies*, **14** (1), 176.

16 Houser, J., Pezeshki, A., Clement, J.T., Aaron, D., Mench, M.M. (2017) Architecture for improved mass transport and system performance in redox flow batteries. *Journal of Power Sources*, **351**, 96–105.

17 Dennison, C.R., Agar, E., Akuzum, B., Kumbur, E.C. (2016) Enhancing Mass Transport in Redox Flow Batteries by Tailoring Flow Field and Electrode Design. *J. Electrochem. Soc.*, **163** (1), A5163-A5169.

18 Milshtein, J.D., Tenny, K.M., Barton, J.L., Drake, J., Darling, R.M., Brushett, F.R. (2017) Quantifying Mass Transfer Rates in Redox Flow Batteries. *J. Electrochem. Soc.*, **164** (11), E3265-E3275.

19 Shah, A.A., Watt-Smith, M.J., Walsh, F.C. (2008) A dynamic performance model for redox-flow batteries involving soluble species. *Electrochimica Acta*, **53** (27), 8087–8100.

20 Zhu, H., Yin, C., Lu, M., Li, Z., Ma, Q., Su, H., Yang, W., Xu, Q. (2024) A critical review on operating parameter monitoring/estimation, battery management and control system for redox flow batteries. *Journal of Energy Storage*, **102**, 114029.

21 Kim, D.K., Yoon, S.J., Lee, J., Kim, S. (2018) Parametric study and flow rate optimization of all-vanadium redox flow batteries. *Applied Energy*, **228**, 891–901.

22 Bard, A.J., Faulkner, L.R., White, H.S. (2022) *Electrochemical methods: Fundamentals and applications*, Wiley, Hoboken, NJ, USA, Chichester, West Sussex, UK.

23 Wang, T., Fu, J., Zheng, M., Yu, Z. (2018) Dynamic control strategy for the electrolyte flow rate of vanadium redox flow batteries. *Applied Energy*, **227**, 613–623.

24 Fu, J., Wang, T., Wang, X., Sun, J., Zheng, M. (2017) Dynamic Flow Rate Control for Vanadium Redox Flow Batteries. *Energy Procedia*, **105**, 4482–4491.

25 Elgrishi, N., Rountree, K.J., McCarthy, B.D., Rountree, E.S., Eisenhart, T.T., Dempsey, J.L. (2018) A Practical Beginner’s Guide to Cyclic Voltammetry. *J. Chem. Educ.*, **95** (2), 197–206.

26 Fabbrizzi, L. (2020) The ferrocenium/ferrocene couple: a versatile redox switch. *ChemTexts*, **6** (4).

27 Astruc, D. (2017) Why is Ferrocene so Exceptional? *Eur J Inorg Chem*, **2017** (1), 6–29.

28 Kortekaas, L., Fricke, S., Korshunov, A., Winter, M., Cekic‐Laskovic, I., Grünebaum, M. (2023) A Digital Blueprint for 3D‐Printing Lab Scale Aqueous and Organic Redox‐Flow Batteries. *Batteries & Supercaps*, **6** (6).

29 Alhammadi, A., Fetyan, A., Agung Susantyoko, R., Mustafa, I., Bamgbopa, M.O. (2025) Understanding characteristic electrochemical impedance spectral data of redox flow batteries with multiphysics modeling. *Journal of Energy Chemistry*, **102**, 329–339.

30 Gaberšček, M. (2021) Understanding Li-based battery materials via electrochemical impedance spectroscopy. *Nature communications*, **12** (1), 6513.

31 Milshtein, J.D., Barton, J.L., Carney, T.J., Kowalski, J.A., Darling, R.M., Brushett, F.R. (2017) Towards Low Resistance Nonaqueous Redox Flow Batteries. *J. Electrochem. Soc.*, **164** (12), A2487-A2499.

32 Forner-Cuenca, A., Penn, E.E., Oliveira, A.M., Brushett, F.R. (2019) Exploring the Role of Electrode Microstructure on the Performance of Non-Aqueous Redox Flow Batteries. *J. Electrochem. Soc.*, **166** (10), A2230-A2241.

33 Laschuk, N.O., Easton, E.B., Zenkina, O.V. (2021) Reducing the resistance for the use of electrochemical impedance spectroscopy analysis in materials chemistry. *RSC advances*, **11** (45), 27925–27936.

34 Leuaa, P., Priyadarshani, D., Tripathi, A.K., Neergat, M. (2019) Internal and External Transport of Redox Species across the Porous Thin-Film Electrode/Electrolyte Interface. *J. Phys. Chem. C*, **123** (35), 21440–21447.

35 Leuaa, P., Priyadarshani, D., Choudhury, D., Maurya, R., Neergat, M. (2020) Resolving charge-transfer and mass-transfer processes of VO2+/VO2+ redox species across the electrode/electrolyte interface using electrochemical impedance spectroscopy for vanadium redox flow battery. *RSC advances*, **10** (51), 30887–30895.

36 Wang, H., Sayed, S.Y., Luber, E.J., Olsen, B.C., Shirurkar, S.M., Venkatakrishnan, S., Tefashe, U.M., Farquhar, A.K., Smotkin, E.S., McCreery, R.L., Buriak, J.M. (2020) Redox Flow Batteries: How to Determine Electrochemical Kinetic Parameters. *ACS nano*, **14** (3), 2575–2584.

37 Narayanan Krishnamoorthy, A., Wölke, C., Diddens, D., Maiti, M., Mabrouk, Y., Yan, P., Grünebaum, M., Winter, M., Heuer, A., Cekic‐Laskovic, I. (2022) Data‐Driven Analysis of High‐Throughput Experiments on Liquid Battery Electrolyte Formulations: Unraveling the Impact of Composition on Conductivity**. *Chemistry Methods*, **2** (9).

38 Zhang, C., Qian, Y., Ding, Y., Zhang, L., Guo, X., Zhao, Y., Yu, G. (2019) Biredox Eutectic Electrolytes Derived from Organic Redox-Active Molecules: High-Energy Storage Systems. *Angewandte Chemie (International ed. in English)*, **58** (21), 7045–7050.

39 Wei, X., Duan, W., Huang, J., Zhang, L., Li, B., Reed, D., Xu, W., Sprenkle, V., Wang, W. (2016) A High-Current, Stable Nonaqueous Organic Redox Flow Battery. *ACS Energy Lett.*, **1** (4), 705–711.

40 Laramie, S.M., Milshtein, J.D., Breault, T.M., Brushett, F.R., Thompson, L.T. (2016) Performance and cost characteristics of multi-electron transfer, common ion exchange non-aqueous redox flow batteries. *Journal of Power Sources*, **327**, 681–692.

41 Milshtein, J.D., Barton, J.L., Darling, R.M., Brushett, F.R. (2016) 4-acetamido-2,2,6,6-tetramethylpiperidine-1-oxyl as a model organic redox active compound for nonaqueous flow batteries. *Journal of Power Sources*, **327**, 151–159.

42 Escalante-García, I.L., Wainright, J.S., Thompson, L.T., Savinell, R.F. (2015) Performance of a Non-Aqueous Vanadium Acetylacetonate Prototype Redox Flow Battery: Examination of Separators and Capacity Decay. *J. Electrochem. Soc.*, **162** (3), A363-A372.

43 Yuan, J., Zhang, C., Zhen, Y., Zhao, Y., Li, Y. (2019) Enhancing the performance of an all-organic non-aqueous redox flow battery. *Journal of Power Sources*, **443**, 227283.

44 Duan, W., Huang, J., Kowalski, J.A., Shkrob, I.A., Vijayakumar, M., Walter, E., Pan, B., Yang, Z., Milshtein, J.D., Li, B., Liao, C., Zhang, Z., Wang, W., Liu, J., Moore, J.S., Brushett, F.R., Zhang, L., Wei, X. (2017) “Wine-Dark Sea” in an Organic Flow Battery: Storing Negative Charge in 2,1,3-Benzothiadiazole Radicals Leads to Improved Cyclability. *ACS Energy Lett.*, **2** (5), 1156–1161.

45 Kwon, G., Lee, K., Lee, M.H., Lee, B., Lee, S., Jung, S.-K., Ku, K., Kim, J., Park, S.Y., Kwon, J.E., Kang, K. (2019) Bio-inspired Molecular Redesign of a Multi-redox Catholyte for High-Energy Non-aqueous Organic Redox Flow Batteries. *Chem*, **5** (10), 2642–2656.

46 Kwon, G., Lee, S., Hwang, J., Shim, H.-S., Lee, B., Lee, M.H., Ko, Y., Jung, S.-K., Ku, K., Hong, J., Kang, K. (2018) Multi-redox Molecule for High-Energy Redox Flow Batteries. *Joule*, **2** (9), 1771–1782.

47 Tracy, J.S., Horst, E.S., Roytman, V.A., Toste, F.D. (2022) Development of high-voltage bipolar redox-active organic molecules through the electronic coupling of catholyte and anolyte structures. *Chemical science*, **13** (36), 10806–10814.

48 Duan, W., Vemuri, R.S., Milshtein, J.D., Laramie, S., Dmello, R.D., Huang, J., Zhang, L., Hu, D., Vijayakumar, M., Wang, W., Liu, J., Darling, R.M., Thompson, L., Smith, K., Moore, J.S., Brushett, F.R., Wei, X. (2016) A symmetric organic-based nonaqueous redox flow battery and its state of charge diagnostics by FTIR. *J. Mater. Chem. A*, **4** (15), 5448–5456.

49 Hudak, N.S., Small, L.J., Pratt, H.D., Anderson, T.M. (2015) Through-Plane Conductivities of Membranes for Nonaqueous Redox Flow Batteries. *J. Electrochem. Soc.*, **162** (10), A2188-A2194.

50 Xing, X., Zhao, Y., Li, Y. (2015) A non-aqueous redox flow battery based on tris(1,10-phenanthroline) complexes of iron(II) and cobalt(II). *Journal of Power Sources*, **293**, 778–783.

51 Xing, X., Huo, Y., Wang, X., Zhao, Y., Li, Y. (2017) A benzophenone-based anolyte for high energy density all-organic redox flow battery. *International Journal of Hydrogen Energy*, **42** (27), 17488–17494.

52 Hagemann, T., Winsberg, J., Häupler, B., Janoschka, T., Gruber, J.J., Wild, A., Schubert, U.S. (2017) A bipolar nitronyl nitroxide small molecule for an all-organic symmetric redox-flow battery. *NPG Asia Mater*, **9** (1), e340-e340.

53 Hu, B., DeBruler, C., Rhodes, Z., Liu, T.L. (2017) Long-Cycling Aqueous Organic Redox Flow Battery (AORFB) toward Sustainable and Safe Energy Storage. *Journal of the American Chemical Society*, **139** (3), 1207–1214.

54 Beh, E.S., Porcellinis, D. de, Gracia, R.L., Xia, K.T., Gordon, R.G., Aziz, M.J. (2017) A Neutral pH Aqueous Organic–Organometallic Redox Flow Battery with Extremely High Capacity Retention. *ACS Energy Lett.*, **2** (3), 639–644.

55 Hoober-Burkhardt, L., Krishnamoorthy, S., Yang, B., Murali, A., Nirmalchandar, A., Prakash, G.K.S., Narayanan, S.R. (2017) A New Michael-Reaction-Resistant Benzoquinone for Aqueous Organic Redox Flow Batteries. *J. Electrochem. Soc.*, **164** (4), A600-A607.

56 Lin, K., Gómez-Bombarelli, R., Beh, E.S., Tong, L., Chen, Q., Valle, A., Aspuru-Guzik, A., Aziz, M.J., Gordon, R.G. (2016) A redox-flow battery with an alloxazine-based organic electrolyte. *Nat Energy*, **1** (9).

57 Janoschka, T., Martin, N., Hager, M.D., Schubert, U.S. (2016) An Aqueous Redox-Flow Battery with High Capacity and Power: The TEMPTMA/MV System. *Angewandte Chemie (International ed. in English)*, **55** (46), 14427–14430.
